# The Form and Function of Retinal Ganglion Cells in Diabetes

**DOI:** 10.3390/cells14181455

**Published:** 2025-09-17

**Authors:** Alistair J. Barber

**Affiliations:** Ophthalmology, and Cell & Biological Systems, Milton S. Hershey Medical Center, Penn State University College of Medicine, Room C4800, Mail code H166, 500 University Drive, Hershey, PA 17033, USA; abarber@psu.edu; Tel.: +1-(717)531-6506; Fax: +1-(717)531-0065

**Keywords:** apoptosis, neurodegeneration, melanopsin, contrast sensitivity, nerve fiber layer, optic nerve

## Abstract

This review examines how diabetes affects the ganglion cells of the retina, including the axons that make up the optic nerve. Links between established changes in the morphology of retinal ganglion cells (RGCs) and vision loss, as well as other functions, such as the pupillary light reflex, are considered. RGC morphology and function are significantly altered in both animal models and humans with diabetes. Diabetes affects all parts of the RGC, including the dendrites, the cell body, the axons making up the nerve fiber layer, and the optic nerve. Subtypes of RGCs appear to be affected differently by diabetes, and the morphology and electrophysiological output are more significantly affected in ON-RGCs than in OFF cells, which may explain part of the mechanism underlying the widely documented diabetes-induced reduction in contrast sensitivity. Furthermore, the morphology of the specialized light-sensitive melanopsin-containing RGCs also appears to be affected by diabetes, which may explain deficits in circadian rhythm and the pupillary light reflex. Potential therapeutic approaches aimed at protecting RGCs in diabetes are also discussed. Overall, strong evidence supports the conclusion that diabetes impacts the form and function of RGCs and their axons within the optic nerve, resulting in deficient regulation of circadian rhythms and the pupillary light reflex, in addition to vision.

## 1. Introduction

The complex pathology of diabetic retinopathy (DR) has been described with increasing detail for many years and includes a variety of vascular lesions, including microaneurysms, neovascularization, acellular capillaries, lipid deposits associated with leaking blood vessels, and macular edema [1,2,3]. The understanding of DR pathology includes observations of damage to neurons, including retinal ganglion cells (RGCs). Early histological studies recorded observations of degenerating or necrotic RGCs, with swollen cell bodies and axons [4,5]. These studies also described RGCs containing “pyknotic” nuclei, a histological term for condensed and fragmented chromatin, which later became recognized as a hallmark of apoptosis [6]. Later histology studies revealed increases in neural apoptosis that led to the depletion of RGCs and other neurons [7]. The fascinating history of the early histological studies of the postmortem retinas of diabetic patients was succinctly reviewed already by Lynch and Abramoff [8]. The overarching conclusion of this and other reviews was that the neurodegenerative components of DR are early changes that may precede the gross retino-vascular pathologies detectible by clinical examination [8,9,10]. However, the mechanisms and functional consequences of neurodegeneration, and in particular the relationship between apoptosis of RGCs and vision loss, remain to be fully determined. Here, I focus specifically on how alterations to the structure and function of RGCs could lead to vision loss and other functional changes associated with diabetes.

## 2. Apoptosis of RGCs and Other Neural Cells in Diabetes

The initial evidence suggesting that diabetes causes the degeneration of cells in the inner retina came from histological studies of cell loss or apoptosis in animal models. Counting the number of large-cell bodies in the ganglion cell layer of H&E-stained radial sections from rats 7–8 months after streptozotocin (STZ)-induced diabetes revealed a 12% reduction in RGCs [7]. The accelerated cell death was confirmed using terminal dUTP nick-end labeling (TUNEL) to identify apoptosis-positive nuclei in rats with short durations of hyperglycemia (2–4 weeks). Due to the low frequency of baseline apoptosis, the total number of TUNEL-positive cells was determined by scanning whole-mount retinas using bright-field microscopy. The number of retinal cells with positive immunoreactivity for the active form of caspase-3 was also increased in STZ-diabetic rats during the first 4 weeks of hyperglycemia, using a similar quantification approach [11]. The positions and antigenicity of caspase-3-positive cells were consistent with bipolar neurons, amacrine cells, and RGCs. Co-localization with NeuN, tyrosine hydroxylase, and choline acetyltransferase further indicated that many apoptotic cells were neurons [12]. Further evidence from other investigators confirmed the loss of RGCs. Quantification of Thy-1-labeled RGCs in 3-month diabetic animals revealed a 16% reduction compared to controls [13]. A similar analysis of flat-mount retinas from spontaneously diabetic Ins2^Akita^ mice with an endogenously fluorescent protein expressed under the Thy-1 promoter also indicated a 16% reduction of RGCs, compared to wild-type controls [14]. Cell loss was mostly in the peripheral retina in this study, while RGCs in the central area of the retina appeared to be spared, at least in mice after 3 months of hyperglycemia. The increase in TUNEL labeling and other apoptosis markers was confirmed in STZ-diabetic mice [15] and models of type 2 diabetes, such as the Otsuka Long–Evans Tokushima Fatty rats (OLETF), which are spontaneously obese rats that develop late-onset hyperglycemia, as well as the Ob/Ob and db/db mouse models, which have leptin signaling defects leading to hyperphagia and dyslipidemia [16,17,18]. Apoptosis was also noted in the postmortem retinas of humans with diabetes [7,19]. Together, these studies provide conclusive evidence that diabetes elevates neuronal apoptosis in the retina and that RGCs are among the cells affected.

Initially, the lack of good immunological markers for RGCs meant that quantification relied heavily on counting large cell bodies in radial sections, with a high probability of sampling errors and inaccurate estimates of total cell death. Immunolabelling of Brn3a, a cell-specific transcription factor, improved RGC quantification [20] and showed that STZ-diabetes significantly reduced RGCs by about 7% after 6 weeks and 15% after 12 weeks of hyperglycemia in rodents [21]. Similarly, a significant decrease in Brn3a-positive cells was measured in the retinas of diabetic rats after 1 week of hyperglycemia [22], and the number of Brn3a-immunoreactive cell bodies was 20% fewer in 9-month-old Ins2^Akita^ mice compared to wild-type controls [23]. This study also examined mice at 3 and 6 months of age and found no difference in cell numbers in the younger diabetic groups, but the quantification was in histological radial sections, rather than whole-mount retinas. Quantification of Brn3a-positive cells in whole-mount retinas found a loss of 8–11% of RGCs, depending upon the region of retina, in 7-month-old Ins2^Akita^ mice [24]. The loss of both RGCs and dopaminergic amacrine cells through apoptosis was confirmed in diabetic rats [25]. A combination of RBPMS and NeuN immunofluorescence indicated a loss of about 18% of RGCs in flat-mount retinas of diabetic mice after 8–10 weeks [26]. Another marker, the RNA-binding protein with multiple splicing (RBPMS), has also been suggested as the preferred method for the quantification of RGCs because this antigen is expressed in the entire population of RGCs [27].

Despite the variations in the estimated number of RGCs lost and the rate at which the cells disappear in different animal models, there is an abundance of data demonstrating a reduction in the number of RGCs due to diabetes. The results establish that RGC loss is a significant consequence of diabetes, beginning soon after the onset of hyperglycemia.

## 3. Mechanisms of RGC Loss in Diabetes

The underlying mechanism of RGC death in diabetes is unclear. It appears, however, that RGC loss follows the canonical apoptosis signaling pathway, including caspase activation, cytosolic cytochrome c accumulation, and indicators of mitochondrial dysfunction [28,29], while autophagy may play a lesser role and could even be cytoprotective to RGCs [30]. One frequently suggested mechanism to explain cell death is oxidative stress, which causes a broad range of damage to cells, including mitochondrial dysfunction [28,31,32]. The impact of diabetes on the mitochondria of various retinal cell types, including vascular endothelial cells, may be key to both cell death and compromised metabolic function [33,34,35]. Oxidative stress is a common factor in diabetic complications, and it may be mediated in RGCs by the expression of the mitochondria-associated adaptor protein, p66Shc, which has a greater expression in the ganglion cell layer, suggesting a higher susceptibility of RGCs [36,37]. Another interesting proposal is that tau hyperphosphorylation through the activation of diabetes-induced GSK3β could disrupt mitochondrial transport and synaptic energy production in neurons, compromising electrophysiological function and leading to neurodegeneration [38]. The impact of hyperglycemia on mitochondrial Ca^2+^ buffering could also explain the abnormal depolarization-induced intracellular Ca^2+^ response in neuronal cultures and retinal tissue slices [39,40]. Stabilizing the mitochondrial electron transport chain with the drug MTP-131 (Elamipretide) protected against diabetes-induced vision loss in mice, further implicating a role for mitochondria in retinal dysfunction [41,42]. Improvement in the vision of db/db mice was also achieved recently by using the short-chain quinone, ubiquinone (CoQ10), administered as eye drops [43]. These data suggest that part of the neuronal dysfunction in DR is due to mitochondrial insufficiencies that may be partially corrected using mitoprotective treatments.

The increase in oxidative stress in diabetes appears aggravated by a reduction in the effectiveness of the oxidative stress response in the retina, and this may have a particular impact on the survival of RGCs. The oxidative stress response is regulated by a key transcription factor, nuclear factor erythroid-2-related factor 2 (Nrf2), which induces several genes that reduce superoxide. A transgenic mutation of Nrf2 in diabetic mice significantly exacerbated the loss of visual function, including contrast sensitivity, in diabetic mice, suggesting that inner-retina neurons were compromised by the loss of Nrf2 [44]. Epigenetic modification of the Nrf2 transcription factor gene and its regulatory partner, KEAP1, may be responsible for the weakening of the oxidative stress response [45,46,47]. Retinal pathology and vision loss have also been prevented by dietary antioxidants that may activate Nrf2, such as astaxanthin and other xanthophylls [48,49,50,51,52].

Other potential mechanisms that trigger RGC apoptosis in diabetes include the O-GlcNAcylation of proteins, such as NF-κB, by increased flux of glucose through the hexosamine biosynthetic pathway [53]. O-GlcNAcylation of other stress-response proteins, such as REDD1, may also play a role in mitochondrial dysfunction, oxidative stress, and translational regulation, potentially inducing apoptosis and a loss of visual function [54,55,56,57].

Glutamate toxicity is another potential mechanism of RGC loss in diabetes [9,58,59,60]. Early studies reported dysregulation of glutamate metabolism, introducing the possibility of chronic glutamate excitotoxicity in diabetes [61,62]. The mechanism of dysregulation is potentially through alterations in the transamination of branched-chain amino acids [63,64,65,66]. Significant changes in the expression of genes related to ionotropic glutamate neurotransmission and transport in STZ-diabetic Long–Evans rats also support the glutamate excitotoxicity hypothesis to explain RGC loss [67].

While the current consensus favors oxidative stress as the most probable cause of RGC death, other factors may contribute to their vulnerability, including their size, their dependence on growth factors, and the loss of input from lower neurons within the retina. For these reasons, RGCs may be more susceptible to diabetes than other retinal cells.

## 4. Thinning of the Nerve Fiber Layer Reflects the Loss of RGCs

The apoptosis-induced loss of RGC cells is likely accompanied by a reduction in the number of axons. Since the RGC axons make up the nerve fiber layer (NFL) in the inner retina, the death of RGCs will be reflected by NFL thinning. Clinical studies have noted that diabetes results in a reduction in the thickness of the NFL, and the most likely interpretation of these observations is the loss of RGC axons [68]. When scanning laser polarimetry was used to measure the NFL [69], the non-diabetic control group’s NFL thickness was 67.7 ± 9.5 µm, while in the diabetic groups with either well-regulated or poorly regulated blood glucose, the NFL thicknesses were 65.1 ± 9.9 µm and 64.1 ± 11.9 µm, respectively. These small but significant reductions in NFL thickness suggested a degeneration that could have broad consequences for the functional output of the retina.

The advent of high-resolution spectral domain–optical coherence tomography (SD-OCT) confirmed and extended the previous clinical findings on thinning of the neural retina. While SD-OCT is used widely for diagnostic purposes to detect retinal swelling due to macular edema, some studies have determined subtle reductions in retinal thickness in diabetic populations with no edema. Retinal thickness in the pericentral area was found to be thinner by an average of 14 µm in patients with type 1 diabetes compared to healthy controls [70]. The patients had minimal DR, suggesting that thinning was due to a loss of intra-retinal neural tissue during the initial stages of disease. Another study using SD-OCT observed a more specific reduction in the thickness of the pericentral and peripheral NFL, as well as reductions in the thickness of the RGC layer and inner plexiform layer (IPL), in patients with type 1 diabetes compared to non-diabetic controls [71]. A series of other clinical studies have reported thinning of various regions of the retina, mostly due to loss of the NFL and the RGC + IPL in both type 1 and type 2 diabetic patients [72,73,74]. In addition to the loss of RGCs, other contributions to thinning of the inner retina may include a reduction in the number of amacrine cells, as demonstrated in several animal studies [12,25,75]. Furthermore, a decrease in bipolar cell dendritic boutons and axon terminals may also contribute to IPL thinning [43,76]. A reduction in input from amacrine and bipolar cells is likely to add to the dysfunction caused by loss of RGCs.

While the diabetes-induced changes in retinal thickness are small in absolute terms, they are typically highly statistically significant. There is a question of whether the small loss of tissue has clinical significance. However, a reduction in the thickness of the inner layers of the retina correlates with loss of visual function, as determined by the Rarebit visual field test, which is sensitive to subtle deficits in the macular visual field [77]. Further studies have confirmed the correlation between a thinning of inner retina structures and a loss of visual function [78,79]. Thinning of the INL also correlates with the presence of peripheral neuropathy, which further suggests that DR has a clinically significant neurodegenerative component [80].

There is now overwhelming evidence that diabetes induces significant thinning of the NFL and other regions of the inner retina, which can be detected soon after the onset of diabetes, often in the absence of vascular lesions [81]. These data have been more effectively reviewed by others [82] and have culminated more recently in rigorous clinical investigations, such as the Maastricht Study, which found that thinning of the NFL occurs even in patients with pre-diabetes [83]. A longitudinal study, which followed the same patients over four years, estimated the rate of loss of inner retina tissue [84]. In this study, diabetes induced a reduction of the NFL thickness by an average of 0.25 µm/year, while the thickness of the combined RGC and IPL decreased at a rate of 0.29 µm/year. A later study followed a group of type 2 diabetic patients over three years and estimated an even greater rate of loss of the peripapillary nerve fiber layer, of 1.34 µm per year [85]. The rate of diabetes-induced inner retina tissue loss appears to be comparable to that estimated for patients with mild-to-moderate primary open-angle glaucoma, which averages between 0.72 µm and 2.08 µm/year, depending on the study [86,87,88,89]. However, the amount of inner retina thinning needed to exceed the threshold for clinically significant vision loss is not well-established. The potential for functional consequences of insidious RGC loss in diabetes suggests that early detection and prevention are important goals for DR research and clinical care. Enhanced imaging, such as high-resolution OCT to measure the disorganization of the retinal inner layers (DRIL), may help in this regard [90].

## 5. Diabetes Alters the Dendritic Field Morphology of RGCs

Before the onset of cell death, neurons undergo degenerative changes that include altered expression of proteins relevant to axonal transport and neurotransmission, as well as modifications in structural morphology [91]. Changes to the structure of RGCs may also occur in response to the loss of input from lower neurons in the retina [92]. The complexity of the three-dimensional structure of RGC dendrites, as well as their overlapping nature, has made the analysis of their morphology technically difficult. However, there have been a small number of studies that determined the impact of diabetes on the morphology of RGCs [93]. One group used gene-gun delivery of the lipophilic fluorophore, DiI, to label individual neurons in the retinas of 3-month STZ-diabetic rats [13]. Using this technique, the entire dendritic structure of random neurons was labeled, revealing abnormal features in some RGCs, including thickening and shortening of dendrites. There was also a significant increase in the dendritic field diameter of a subset of RGCs with large cell bodies. Morphological abnormalities were also noted in DiI-labeled midget and parasol RGC dendrites in the postmortem retinas of a small number of diabetic patients [94].

**Figure 1:**
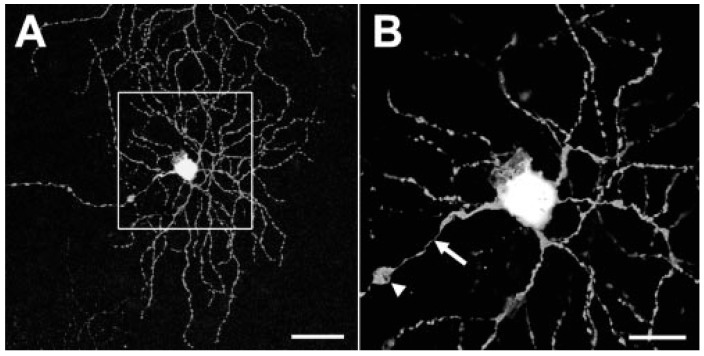
RGC cell body and axon swelling in diabetic mouse retinas. The effect of diabetes on the morphology of RGCs was explored by crossing Thy1-YFP and Ins2^Akita/+^ mice and imaging flat-mount retinas by confocal microscopy. (**A**) The entire structure of individual RGCs, including axon, cell body, and dendritic fields, could be viewed (scale bar represents 50 µm); (**B**) axon swellings (arrowhead) often appeared close to the cell body, associated with narrowing closer to the cell body (arrow; scale bar represents 20 µm). (Reproduced from [14]. The author acknowledges the Association for Research in Vision and Ophthalmology as the copyright holder).

**Figure 2:**
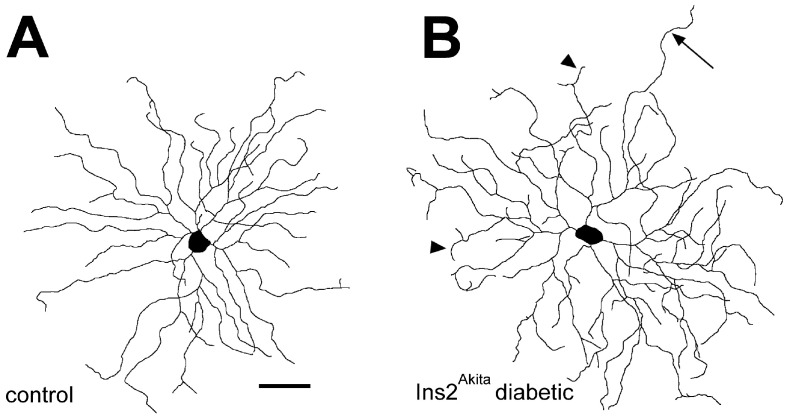
Dendritic structure of large ON-type RGCs in diabetic mouse retina. The effect of diabetes on the dendritic structure of RGCs was explored by making line tracings of Thy1-YFP-positive cells imaged by confocal microscopy in flat-mount retinas. (**A**) Wild-type (control) and (B) Ins2^Akita/+^ mice after three months of hyperglycemia. The dendritic field of large ON-type RGCs was significantly denser in the Ins2^Akita/+^ mice compared to controls (measured by Sholl analysis). Terminal dendrites often appeared truncated or bifurcated (arrowheads in (**B**) and sometimes were unbranched and extended beyond the normal dendritic arbor (large arrow in (**B**)). Scale bar represents 50 µm. (Reproduced from [14]. The author acknowledges the Association for Research in Vision and Ophthalmology as the copyright holder).

To further examine the dendritic structure and abundance of RGCs in diabetic mouse retinas, Thy-1-YFP and -CFP transgenic mice were crossed with Ins2^Akita^ mice to generate a genetic model of diabetes with endogenously fluorescent RGCs [11,14]. Diabetes diminished the number of Thy-1-CFP fluorescent cells by 16%, confirming earlier quantification studies. Furthermore, abnormal swellings appeared on the axons of some Thy-1-YFP positive RGCs, often accompanied by nearby axon thinning, possibly indicating axoplasmic transport dysfunction (Figure 1) [14]. Scholl analysis revealed that the number of dendritic terminals and dendrite density of ON-RGCs were increased in diabetic mice by 32.4% and 18.6% respectively, while the total dendritic length was increased by 15.3%, compared to age-matched controls. Notably, the changes in dendrite morphology were primarily in the ON-RGCs that possessed large cell bodies, suggesting greater vulnerability of this subtype (Figure 2).

The diabetes-induced increase in dendrite complexity of some RGCs may be compensatory to a loss of inhibitory input from parallel processing neurons such as amacrine cells. This possibility is supported by several morphological studies reporting the depletion of a variety of amacrine cell subtypes in diabetic animal models [12,23,75,95,96,97]. A further possibility is that there is a broad reduction in synaptic input to RGCs caused by a downregulation of presynaptic neurotransmitter release mechanisms within the inner plexiform synapses [23,98,99,100]. This concept is also supported by the results of a proteomic analysis of postmortem human retinas showing an enrichment of signaling pathways associated with synaptic long-term potentiation mechanisms, dopamine pathway degradation, and other neurodegenerative processes [101]. The consensus from studies of the effect of diabetes on RGC dendrite morphology strongly suggests that plastic changes occur at the dendritic and synaptic levels in response to the loss of input from other neurons.

## 6. RGC Pathology Reduces the Scotopic Threshold Response

The traditional electroretinogram (ERG) is well-established to be altered in several ways by diabetes [102], but it does not register the electrophysiological signal of the RGCs because their combined electrical output is too weak compared to the collective signal from the rest of the retina, especially in response to bright stimuli. The small positive scotopic threshold response (pSTR) appears in the ERG recording around 200 milliseconds after a low-energy flash of light. The pSTR is thought to detect only RGC and amacrine cell depolarizations and is reduced by optic nerve transection, while the a and b waves of the normal ERG response are unaffected [103]. Studies have reported a significantly smaller amplitude in the pSTR waveform in STZ-diabetic rodents, by as much as 50% in some cases, and the deficit became significant within 4 weeks of hyperglycemia, while the a- and b-wave components of the ERG were unaffected [104,105,106]. Similarly, diabetic Ins2^Akita^ mice had a significantly lower pSTR amplitude after three months of diabetes [107,108]. The data from diabetic animals confirmed earlier clinical reports of significant pSTR deficits in patients who had diabetes without visible signs of vascular retinopathy [109,110]. The diminished pSTR suggests that diabetes reduces the amplitude of the RGC electrophysiological output soon after the onset of hyperglycemia compared to other changes in the retina.

## 7. Diabetes Alters the Pattern-ERG Response

The pattern ERG (pERG) is a sensitive focal electrophysiology technique designed to measure the functionality of RGCs. The pERG uses an alternating grating stimulus rather than a simple flash of light and can therefore be considered a measure of inner retina information processing (reviewed in [111]). Specifically, the pERG detects RGC output because optic nerve transection causes the progressive disappearance of the pERG signal, while the regular flash-response ERG remains intact [112]. pERG deficits in db/db and STZ-diabetic mice have been reported [18,113,114]. The pERG and visual-evoked potential were also reduced by a diabetogenic high-fat diet in mice, in the absence of retinal vascular abnormalities, and this was associated with hyperphosphorylation of tau in both the retina and optic nerve, suggesting that this model shares features common to other chronic neurodegenerative diseases [38]. The deficit in pERG in diabetic rodents may be related to oxidative stress because it was corrected by the inhibition of NOX4 [115].

Diabetes also significantly reduces the pERG in humans, even in the absence of vascular retinopathy or reduction in visual acuity, but corresponds to reductions in contrast sensitivity [116,117]. Several studies of patients with insulin-dependent diabetes have detected inner retina dysfunction using the pERG [111,118,119]. The amplitude of the RGC signal detected by pERG was significantly lower, even in patients with no visible retinovascular changes, and appeared to worsen with a longer duration of diabetes [120,121,122,123]. At the onset of vascular retinopathy, and once patients have developed significant vascular DR, the severity of the pERG deficit increases [124]. There are, however, some clinical studies that found no deficits in pERG in diabetic patients with no vascular retinopathy [125,126]. Other studies have found significant correlations between the pERG deficit and superficial capillary density detected by OCT-A, as well as vascular dilation induced by flicker, suggesting a link between vascular deficits and RGC function [125,127,128].

Data generated by pERG recording provide further evidence that the output of RGCs is significantly degraded by diabetes in both humans and animals. It may be possible, after further validation, that the pERG can be adopted as a predictive diagnostic for the progression of retinal neurodegeneration and vision loss in diabetes.

## 8. Diabetes Alters the Single-Cell Electrophysiology of RGCs

Alterations to the morphology of RGCs, especially the structure of the dendritic field, are likely to be accompanied by changes in the electrophysiological behavior of individual neurons. Several electrophysiological studies have attempted to rigorously explore this possibility, despite the technical challenges of single-cell recordings in the retina. In isolated RGCs, the depolarization-induced firing frequency was reduced in cells from 2-month STZ-diabetic rats compared to controls, while the stimulation-induced calcium signal was increased [129]. Similarly, dissociated RGCs from diabetic rats had a lower sodium and potassium current density [130]. These data suggest that the electrophysiological characteristics of individual RGCs are significantly altered by diabetes. Although these studies did not recapitulate the intact retina, they indicate an intrinsic change in RGC membrane physiology induced by diabetes.

Single-cell patch-clamping has been used to further understand how diabetes impacts the response of RGCs in intact retinas. Diabetes significantly elevated the spontaneous spiking activity of the ON-type RGCs in flat-mount retinas excised from mice after 3–4 months of STZ-induced hyperglycemia, compared to other types of RGCs [131]. The increase in ON-RGC activity was accompanied by a reduced inhibitory input. Another study showed that a diabetes-induced reduction of inhibitory input was linked to the slowing of the calcium response in GABAergic amacrine cells lateral to the rod bipolar cells, suggesting a mechanism for some of the changes at the RGC level that involves deficits in their regulatory input [132].

An elegant approach to recording the light-induced responses of single RGCs in the whole retina used eye cups excised from db/db mice, stimulated by computer-generated images displayed on a small CRT monitor with a first-surface mirror and lens positioned at the film plane of a microscope camera port [133]. Using this approach, Xiao et al. demonstrated that the contrast gain in response to a sinusoidal grating stimulus was significantly reduced in both ON- and OFF-RGCs in the retinas of the db/db mice compared to controls [134]. Furthermore, the average ON-RGC receptive field diameter was significantly reduced after 12 weeks of diabetes. The average receptive field diameter of the OFF-RGCs was also reduced, but not until later, at 20 weeks of diabetes. The luminance threshold of ON-RGCs was also significantly elevated by diabetes, while it remained unaltered in the OFF-RGCs, indicating a differential loss of sensitivity between ON- and OFF-RGCs. In another study, excised wild-type mouse retinas incubated in high glucose media or mannitol also reduced the receptive field size of ON-RGCs and attenuated their contrast gain, suggesting that osmotic changes may be responsible for some of the electrophysiological abnormalities, although osmotic pressure alone cannot explain the differential deficits between the ON and OFF-RGC responses [135]. Another study confirmed that ON-RGCs in diabetic mice preferentially have an increase in resting membrane potential with decreased membrane capacitance, compared to the OFF-RGCs, and that the electrophysiological changes are accompanied by an altered morphology [136]. More recently, single-cell recording of RGCs in explant retinas stimulated with green light also revealed a preferential deficit in the ON-alpha RGCs that is not recapitulated in the OFF-RGCs [137]. Since increases in the spontaneous activity of RGCs are indicative of retinal neurodegeneration in other models [138], the electrophysiological data from diabetic animals further support retinal neurodegeneration in DR. Also, diabetes increased light-evoked excitatory post-synaptic potentials of ON-RGCs in both light and dark conditions, suggesting that they receive excessive excitation [139]. The impaired light adaptation of the ON-RGCs could be through a diabetes-induced reduction in dopamine D4 receptor activation [140], and there have been several suggestions that retinal dopaminergic signaling is altered by diabetes [141,142,143], which could explain some of the early visual deficits, as well as abnormalities in the oscillatory potentials of the regular ERG.

The combined evidence from STR, pERG, and single-cell recording studies indicates that diabetes causes a signal-processing deficit that preferentially alters the ON-RGC pathway more than the OFF pathway. The impact on the ON-RGCs suggests a potential mechanism for the functional differences in contrast sensitivity that are widely observed in diabetes because this feature of vision is a product of confluent excitatory signals from the ON and OFF pathways [144,145]. It is unclear why ON-RGCs appear to be more affected compared to the OFF cells, but the functional deficit may be partially due to reduced inhibitory input from amacrine cells, leading to an imbalance of input from ON and OFF bipolar cells [132,146]. A potential mechanism for this deficit is a reduction in calcium permeability of amacrine AMPA and GABA receptors, leading to the dynamic dysfunction of inner retinal microcircuitry, theorized to increase glutamate release from bipolar cells onto RGCs [147,148]. This change may be an exaggeration of the established intrinsic asymmetry between the ON-RGC and OFF-RGC pathways [149,150]. See [151] for an excellent review of this theory. It is interesting to note that, in glaucoma models, another disease affecting the survival of neurons in the inner retina that causes a reduction in the NFL thickness, there is also differential susceptibility of RGC subtypes [152,153]. Overall, diabetes alters RGC electrophysiology in ways that could explain early symptoms of vision loss, such as the well-established deficits in contrast sensitivity.

## 9. Diabetes Causes Pathological Changes Within the Optic Nerve

The optic nerve is comprised in part by the axons of RGCs, which are arguably the largest component of these neurons because of their length. The RGC axons begin at the cell body and project across the inner surface of the retina, forming the NFL, before turning through 90° at the optic disc and traversing the lamina cribrosa, where they form the optic nerve, which projects posteriorly towards the optic chiasm. The axons decussate either ipsilaterally or contralaterally to form the optic tracts, and in humans, they synapse predominantly in the lateral geniculate nucleus of the thalamus. A smaller number of RGC axons terminate in other brain regions, such as the superior colliculus and the suprachiasmatic nucleus. The optic nerve is myelinated similarly to peripheral nerves, although the myelination is derived from oligodendrocytes rather than Schwann cells, as with other cranial nerves that are continuous with the meninges. In adult humans, the optic nerves are likely to be between 35 and 55 mm long, and about 15 mm in an adult mouse [154]. These long axons arguably comprise the largest component of the neuron, yet the impact of diabetes on the optic nerves has been largely ignored. This is despite work on glaucoma showing that the optic nerve is affected in parallel with the pathological changes that take place in the retina [155].

The evidence that diabetes impacts RGC axons includes studies quantifying the fibers of the optic nerve, which can be achieved using immunological markers such as neurofilament. Early histological studies established a reduction in the number of optic nerve axons in diabetic rats [156] and a significant (40%) loss of axons in the optic nerves of dogs after 5 years of diabetes [157]. The reduction in axons was interpreted as suggesting RGC loss, although the cell bodies in these studies were not quantified. An in vivo proton MRS study of diabetic rats identified metabolic changes in the visual cortex [158]. Other pathologies included reduced fiber bundles, loss of myelin, and axonal degeneration, which were revealed by electron microscopy. Reduced retrograde fluorogold labeling in the retina after intracerebral injections was initially interpreted to indicate an unexpectedly high loss of RGCs, but several studies demonstrated that dysfunctional retrograde axoplasmic transport was responsible for the failure of fluorogold to reach the RGC cell bodies in diabetic animals [159,160,161,162]. Six weeks of STZ-diabetes in rats also reduced the amount of anterograde transport to the superior colliculus, as well as the axon number and amount of phosphorylated heavy neurofilament in the optic nerve, along with increased reactivity of microglia and astrocytic expression of GFAP in the distal optic nerve [163,164]. These changes occurred despite a lack of significant RGC loss after this short duration of diabetes, confirming that deficits in axoplasmic transport are likely to precede RGC apoptosis. Similarly, distal retrograde transport was reduced in the Ins2^Akita^ mouse model, preceding significant RGC loss [165]. There have been similar reports of axoplasmic transport deficits in other nerves, such as the sciatic nerve and dorsal root, in models of diabetic neuropathy, suggesting that this loss of function in diabetes may be general to other neurons with long axons [166,167,168,169].

Other histological studies have noted lesions of the optic nerve in STZ-diabetic rats, which include atrophy of nerve fibers with neurite swelling and loss of myelin, as well as astrocytic proliferation, reduced blood flow, and increased permeability of the microvasculature [170]. There are also metabolic changes resulting in galactitol accumulation, which indicates polyol pathway activity in the optic nerve, similar to the retina [171]. STZ-induced diabetic mice with knockdown of the neuroinflammation-modulating lectin, galectin-3, had a reduced expression of inflammatory markers, such as GFAP and microglial reactivity in the optic nerve, compared to the diabetic wild-type mice [172]. There were also more myelinated fibers in the distal optic nerve of the diabetic galectin-3 knock-down mice, suggesting that diabetes induces an inflammatory degenerative process in the optic nerve, similar to that observed in the retina. Another study provided evidence of a reduction in the abundance of antioxidant enzymes, as well as significant decreases in the Na^+^/K^+^-ATPase activity in the optic nerve and visual cortex of diabetic rats [173]. Recent work has suggested that oxybutynin may be an effective treatment for peripheral neuropathy, and future studies could also assess its effect on diabetic optic nerve axoplasmic transport and dysfunction [174].

The data show that the number of optic nerve axons is reduced by diabetes and that axoplasmic transport is slowed, concomitantly with pathological events in the retina. These changes could be an indirect consequence of apoptosis and other pathologies occurring at the level of the RGC cell bodies, but the early onset implies that they are caused by a direct effect of the diabetic physiology working at the local level along the RGC axon.

## 10. Diabetes Compromises the Function of Intrinsically Photosensitive RGCs

The combination of RGC loss and functional deficits within synapses and other components may lead to dysfunction, aside from the acquisition and transmission of visual information. The retina is responsible for other light-induced functions apart from vision, which include regulating circadian rhythms and the pupillary light reflex (PLR). The PLR controls the diameter of the pupil in response to changes in light intensity. The pupil constricts in response to light through contraction of the iris sphincter muscle, and conversely, a reduction in light intensity causes radial muscle contraction. The PLR regulates the amount of light entering the eye and must operate rapidly to compensate for changes in ambient illumination. The reflex is initiated by the intrinsically photosensitive RGCs (ipRGCs), which express the photosensitive pigment melanopsin [175]. There is also indirect rod and cone photoreceptor input to these cells [176]. While ipRGCs have at least six subtypes (M1–M6), only the M1 cells, which express melanopsin to the greatest extent, appear to be involved in the PLR [177]. ipRGCs project axons through the olivary pretectal nucleus to regulate the PLR. They also project to the suprachiasmatic nucleus, where they are thought to orchestrate circadian rhythms [178].

**Figure 3:**
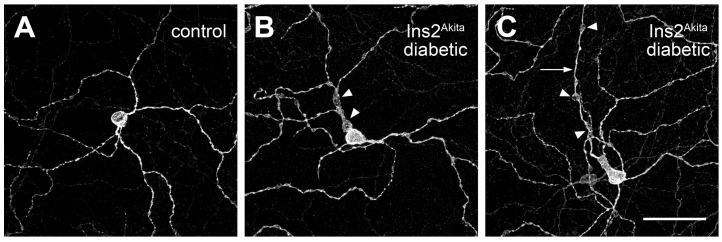
Morphological changes in the ipRGCs of diabetic mice. The effect of diabetes on the morphology of the ipRGCs was determined by confocal microscopy in flat-mount retinas. (**A**) Wild-type (control) and (**B**,**C**) Ins2^Akita/+^ mice labeled by immunofluorescence for melanopsin. Primary dendrites of ipRGCs in diabetic mice were often swollen close to the soma (arrowheads in (**B**)); ipRGC axons in diabetic mice (arrow in (**C**)) also contained multiple swellings (arrowheads in (**C**)). Scale bar represents 50 µm. (Reproduced from [14]. The author acknowledges the Association for Research in Vision and Ophthalmology as the copyright holder).

Scattered evidence suggests that the ipRGCs are impacted by diabetes. The melanopsin-immunoreactive cells of the diabetic Ins2^Akita^ mouse retina were noted to have dendritic and axonal swellings, similar to those observed on other RGCs (Figure 3) [14]. A more detailed morphological study found that the M2 and M3 iPSCs had increased dendritic branching and enlarged soma in STZ-diabetic mice [179]. Dendritic varicosities and soma swelling of ipRGCs were also observed in STZ-diabetic mice after 12 weeks of hyperglycemia, along with a significant reduction in light-induced cFos and circadian clock gene expression in the suprachiasmatic nucleus [180,181]. Although an earlier study showed that 15 weeks of hyperglycemia in STZ-diabetic rats did not change the number of melanopsin-positive RGCs, and a reduction in light-induced cFos expression in the suprachiasmatic nuclei could be reversed by surgical removal of the lens, suggesting that diabetic cataract formation may be partly responsible for changes in the melanopsin-mediated pathway [22]. However, changes in the PLR in STZ-diabetic mice included more rapid pupil constriction and slowed dilation, coinciding with greater amounts of melanopsin mRNA in mice that were diabetic for only 21 days, suggesting that short durations of hyperglycemia cause acute changes in the regulatory mechanisms of the PLR [182].

Clinical studies have established that the PLR becomes compromised in patients with diabetes. PLR dysfunction occurred in a group of adults with insulin-dependent diabetes, and the severity was closely correlated with disease duration. In this study, the PLR parameters that were significantly altered due to diabetes were maximal pupillary area, contraction velocity, and dilation velocity [183]. The degenerative effect of diabetes on the PLR may have an early and rapid onset because adolescents with type 1 diabetes were also found to have significant worsening (more than 60%) of the PLR deficits over 3.5 years [184]. The post-illumination pupil response was also slowed in patients with type 2 diabetes and deteriorated further with longer durations of disease [185]. However, another study found that PLR dysfunction correlated more significantly with the severity of vascular DR rather than the duration of diabetes [186]. The PLR was also impaired in patients with type 2 diabetes, even in those without other autonomic neuropathies or the presence of vascular retinopathy, further suggesting that loss of the PLR has an early onset [187,188,189]. With the development of effective technology, the PLR deficit in diabetes could be easily measured in the clinic, and it has been suggested that it could become an early diagnostic parameter [190].

Taken together, there is compelling evidence that diabetes causes functional changes to the ipRGCs of the retina, detectable as morphological abnormalities, as well as deficits in the PLR and synchronization of circadian rhythms [180,191]. It is unclear, however, what part of the PLR is most impacted by diabetes. The reflex runs through neural relays with four sets of synapses in brain regions outside the retina, and its speed and amplitude are determined under normal circumstances by the efficiency of both contralateral and ipsilateral relays. Therefore, it is possible that diabetes could impair the PLR by affecting neurons and synapses beyond the retina, as well as the ipRGCs within the retina.

## 11. Neuroprotection of RGCs

The diabetes-induced mechanism of RGC dysfunction leading to apoptosis remains unclear, and neuroprotective pharmaceuticals are still in their infancy. Nutraceutical approaches appear to be popular, presumably due to their cost and general focus towards preventing inflammation and oxidative stress. Extracts from the Korean herb *Litsea japonica* have been reported to reduce RGC apoptosis when given to type 2 diabetic db/db mice [192]. The *L. japonica* treatment reduced the accumulation of advanced glycation end-products (AGEs) and the AGE receptor (RAGE) expression in the inner retinas of these mice, accompanied by a significant reduction in the number of TUNEL-positive cells in the RGC layer. Since NF-κB DNA binding was also reduced, the authors suggested that part of the protective mechanism was through the reduction of inflammatory signaling. Another study evaluated a Chinese herbal medicine called He-Ying-Qing-Re formula (HF), which is a combination of eight different herbal sources. In this case, a 4-week treatment of STZ-diabetic mice with HF supplement protected RGCs as much as insulin. HF also partially prevented the diabetes-induced reduction in thickness of the inner plexiform and nuclear layers of the retina, suggesting a broad protection against retinal neurodegeneration [193]. There is significant difficulty in interpreting his type of study, however, because it is often unclear which ingredients are active within the treatment cocktail, leaving doubt as to how to replicate the results using a more targeted pharmaceutical approach.

Other approaches to reduce oxidative stress have focused on dietary compounds that target Nrf2, a key regulator of the oxidative stress response [194,195]. Nrf2 is a central factor in the regulation of oxidative stress in DR and has become a prominent pharmacological target to treat DR, using nutraceuticals or other more conventional pharmacological approaches [44,196,197]. Several studies of dietary pigments, zeaxanthin, astaxanthin, and other carotenoids, report reductions in the markers of metabolic dysfunction and improved retinal electrophysiology [198]. Other studies also found antioxidants to be effective in protecting retinal components, in addition to the RGCs and other neurons [199,200]. Dietary astaxanthin reduced RGC loss in db/db mice [50], and when astaxanthin was fed to the spontaneously diabetic *Psammomys obesus* desert gerbil, the markers of neural dysfunction, such as glutamine synthetase expression, were rectified [52]. Other putative antioxidants suggested to reduce RGC apoptosis include crocin, which is extracted from saffron, and curcumin, extracted from turmeric [201,202]. Another approach to prevent oxidative stress is the elevation of heme oxidase-1 by treatment with hemin. This approach significantly protected RGCs in STZ-diabetic rats, presumably by elevating Nrf2 expression [197,203].

Growth factors could be used to reduce RGC loss. A drug called compound 49b was shown to preserve the number of cells in the RGC layer in diabetic rats, possibly by elevating insulin-like growth factor binding protein, although there may be other targets [204]. Ciliary neurotrophic factor (CNTF) has been suggested as a potential therapeutic that could support RGC survival. STZ-diabetic rats that received intraocular injections of CNTF appeared to have reduced RGC and dopaminergic amacrine cell apoptosis, measured by TUNEL and tyrosine hydroxylase immunolabeling [25]. Also, stimulating the angiotensin-converting enzyme (ACE) pathway, using an ACE2 activator, rescued RGCs in the diabetic rat model [205]. Topical administration of glucagon-like peptide (GLP-1) receptor agonists also rectified neurodegeneration markers in db/db mice that included retinal apoptosis, functional changes in the ERG, and cAMP content in RGCs and other neurons [206]. Meanwhile, pigment epithelium-derived factor (PEDF) has also been shown to protect RGCs in Ins2^Akita^ mice [207]. Brain-derived neurotrophic factor has also received attention as another growth factor affected by diabetes [208,209]. Retinal insulin signaling may also play a role in retinal cell survival and neuroprotection [210,211], by acting through the canonical Akt pathway to promote cell survival [212]. This hypothesis was supported recently by the results of a study that selectively blocked central insulin receptor signaling, leading to RGC apoptosis and optic nerve pathology, along with elevation of inflammatory markers [213]. Topical administration of nerve growth factor (NGF) to Ins2^Akita^ mice prevented RGC loss and inner-retina degeneration, as well as rectifying the ERG [108,214]. The anti-epilepsy drug carbamazepine has also been suggested to be neuroprotective by acting through the TrkA receptor to augment NGF signaling in diabetic mice, thus mimicking NGF signaling [215]. Similarly, the novel microneurotrophin BNN27 activated the TrkA and p75 NGF receptors in the retina by topical or intraperitoneal administration in STZ-diabetic rats and reduced retinal markers of inflammation and neurodegeneration [216,217]. Finally, pharmacological augmentation of growth factors, including NGF signaling, offers several potential agents to treat diabetic retinal degeneration and other neurodegenerative diseases [218].

Glutamate is the most abundant neurotransmitter in the retina, and its levels are influenced by diabetes, along with dysregulation of the glutamate/glutamine cycle, suggesting the possibility that RGCs are affected by glutamate excitotoxicity [63,219,220]. Altering the glutamate cycle with gabapentin may be an alternative approach to protect RGCs from glutamate excitotoxicity in DR [220]. It has also been suggested that chronic treatment with the weak glutamate receptor antagonist, memantine, can prevent glutamate toxicity in diabetic rats [221]. Upregulation of glutamate transport has also been proposed as a method to reduce glutamate excitotoxicity and RGC apoptosis in the retina [222,223]. NADPH oxidase inhibition has also been suggested as a method to reduce both oxidative stress and glutamate excitotoxicity in DR [224].

Tauopathy is strongly associated with neurodegenerative diseases and may be an accompanying pathology in the diabetic retina and optic nerve that could threaten RGC survival. Hyperphosphorylation of tau was identified in high-fat diet-fed mice in both the RGCs and optic nerve [38]. Deficits in the visual-evoked potential in these mice could be corrected by intraocular injection of siRNA for tau, suggesting a link between tauopathy and RGC function and a potential therapeutic target to treat neurodegeneration in DR. In a follow-up study, the same group demonstrated the potential for the GLP-1 receptor agonist, liraglutide, to prevent tau hyperphosphorylation and reduce the amplitude of the visual-evoked potential in high-fat-fed mice [225]. Liraglutide was administered by topical eye drops in this study, as a more direct way to treat the retina and to avoid potential systemic effects on glycemic levels.

While neuroprotection for DR may be in its infancy, lessons can be taken from recent pharmaceutical approaches to prevent RGC death in glaucoma, independently of interventions to reduce intraocular pressure [226]. Clinical trials with supplements such as citicoline, nicotinamide, and pyruvate have shown promising results [227,228,229,230]. The α2-adrenergic agonist, brimonidine, either alone or in combination with other drugs, has also been widely discussed as a candidate neuroprotective therapy in glaucoma [231,232]. In light of glaucoma studies, the efficacy of brimonidine to protect the retina from diabetes was tested in a multicenter clinical trial. The European Consortium for the Early Treatment of Diabetic Retinopathy (EUROCONDOR) focused on neuroprotection using brimonidine and somatostatin eye drops to treat a population of patients with type 2 diabetes [233]. In this study, there was no overall protective effect of either drug. However, in a subset of patients with established retinal dysfunction at baseline, indicated by delayed multifocal ERG implicit time, both drugs significantly prevented the continued loss of function compared to the placebo, over 2 years [234]. The neuroprotective effects of brimonidine were confirmed in STZ rats, even by topical administration [235]. These results suggest that it may be possible to at least slow neurodegeneration and loss of vision if the right treatment is established early in the disease. Further clinical studies using visual function and inner retina thinning as endpoints are needed to better establish neuroprotection as a valid strategy to treat DR.

## 12. Conclusions

A previous review concluded that diabetes causes dysfunction and neurodegeneration of RGCs, but that critical information regarding changes to the anatomy and response properties of RGCs was missing, and the relationship between RGC pathology and visual dysfunction was poorly understood [93]. Here, I attempted to show that the RGC pathology induced by diabetes is now closer to being more completely understood. There is a more comprehensive understanding of how subsets of RGCs are affected by diabetes, including a differential impact on the ON-RGCs more than OFF cells, and that the pathology includes ipRGCs, leading to appreciable deficits in the PLR and circadian rhythms, in addition to the effect on vision.

There is a substantial weight of evidence supporting the hypothesis that degenerative apoptosis of RGCs is a significant consequence of diabetes and that the degeneration begins soon after the onset of hyperglycemia. Careful consideration of the older literature on DR, however, reveals that there is nothing particularly new about the basic observation of RGC pathology in diabetes. In his famous histology study, “Diabetic Retinopathy” published in 1961 in the *American Journal of Ophthalmology*, Professor Reimer Wolter described numerous pathological changes in the retina, including features noted in RGCs, and commented: “*The first change to occur in the retina in diabetes, as far as I know, is the swelling and degeneration of retinal neurons. This involves mainly the neurons of the inner retina layers with all their processes… the primary retinal pathology in diabetes mellitus seems to be neuronal*” [4,236].

The clinical implications of functional deficits and RGC loss are important to consider. Progressive loss of RGCs, even if the number of cells lost is relatively small, will ultimately result in clinically significant vision loss. However, the number of cells that must be lost to cause a detectible deficit in function has not been determined. This “lesion threshold” defines the amount of damage that must occur to cause a deficit that can be detected clinically [236,237]. The clinical result of RGC loss will also be determined by the region of the retina and the type of cell affected. Evidence also strongly suggests that diabetes has a significant impact on RGC structure and function, well before apoptosis causes the cell to be lost. This suggests that the successful therapeutic strategies of the future may be to normalize RGC function, in addition to preventing cell death.

There are numerous hypotheses for the biochemical or metabolic mechanisms responsible for retinal neurodegeneration in diabetes. They include oxidative stress, accumulation of advanced glycation end products, deranged protein glycosylation, glutamate excitotoxicity, chronic subthreshold hypoxia, loss of metabolic support from neighboring glial cells, loss of neurotrophic support and signaling, neuroinflammation, dysfunctional axoplasmic transport, and more. These hypotheses must be tested as rigorously as possible, using a diversity of animal models, to develop a better understanding of the biochemical events that cause the loss of RGCs and other retinal neurons. Finally, more studies must aim to understand the impact of diabetes on the electrophysiology of individual RGCs and the intact optic nerve, and how synaptic input, signal processing, and excitatory dynamics are altered by diabetes, using intracellular recording, patch clamping, and calcium imaging in intact retinas whenever possible.

The estimated incidence of diabetes in the USA has increased to over 14% of the population (CDC National Diabetes Statistics Report, May 2024) and afflicts at least 830 million people worldwide (World Health Organization, November 2024). Given these alarming statistics, the urgency to understand the mechanisms of vision loss and other complications of diabetes has never been greater.

## Data Availability

No new data were created or analyzed for this article, so data sharing is not applicable.

## References

[1] Friedenwald J.S. (1950). Diabetic retinopathy. Am. J. Ophthalmol..

[2] Friedenwald J., Day R. (1950). The vascular lesions of diabetic retinopathy. Bull. Johns Hopkins Hosp..

[3] Engerman R.L., Bloodworth J.M. (1965). Experimental diabetic retinopathy in dogs. Arch. Ophthalmol..

[4] Wolter J.R. (1961). Diabetic retinopathy. Am. J. Ophthalmol..

[5] Bloodworth J.M. (1962). Diabetic retinopathy. Diabetes.

[6] Kerr J.F., Wyllie A.H., Currie A.R. (1972). Apoptosis: A basic biological phenomenon with wide-ranging implications in tissue kinetics. Br. J. Cancer.

[7] Barber A.J., Lieth E., Khin S.A., Antonetti D.A., Buchanan A.G., Gardner T.W. (1998). Neural apoptosis in the retina during experimental and human diabetes. Early onset and effect of insulin. J. Clin. Investig..

[8] Lynch S.K., Abramoff M.D. (2017). Diabetic retinopathy is a neurodegenerative disorder. Vision Res..

[9] Barber A.J. (2003). A new view of diabetic retinopathy: A neurodegenerative disease of the eye. Progress. Neuro-Psychopharmacol. Biol. Psychiatry.

[10] Barber A.J. (2015). Diabetic retinopathy: Recent advances towards understanding neurodegeneration and vision loss. Sci. China Life Sci..

[11] Barber A.J., Antonetti D.A., Kern T.S., Reiter C.E., Soans R.S., Krady J.K., Levison S.W., Gardner T.W., Bronson S.K. (2005). The Ins2Akita Mouse as a Model of Early Retinal Complications in Diabetes. Investig. Ophthalmol. Vis. Sci..

[12] Gastinger M.J., Singh R.S., Barber A.J. (2006). Loss of Cholinergic and Dopaminergic Amacrine Cells in Streptozotocin-Diabetic Rat and Ins2Akita-Diabetic Mouse Retinas. Investig. Ophthalmol. Vis. Sci..

[13] Qin Y., Xu G., Wang W. (2006). Dendritic abnormalities in retinal ganglion cells of three-month diabetic rats. Curr. Eye Res..

[14] Gastinger M.J., Kunselman A.R., Conboy E.E., Bronson S.K., Barber A.J. (2008). Dendrite remodeling and other abnormalities in the retinal ganglion cells of Ins2 Akita diabetic mice. Investig. Ophthalmol. Vis. Sci..

[15] Martin P.M., Roon P., Van Ells T.K., Ganapathy V., Smith S.B. (2004). Death of retinal neurons in streptozotocin-induced diabetic mice. Investig. Ophthalmol. Vis. Sci..

[16] Yang J.H., Kwak H.W., Kim T.G., Han J., Moon S.W., Yu S.Y. (2013). Retinal Neurodegeneration in Type II Diabetic Otsuka Long-Evans Tokushima Fatty Rats. Investig. Ophthalmol. Vis. Sci..

[17] Lee V.K., Hosking B.M., Holeniewska J., Kubala E.C., Lundh von Leithner P., Gardner P.J., Foxton R.H., Shima D.T. (2018). BTBR ob/ob mouse model of type 2 diabetes exhibits early loss of retinal function and retinal inflammation followed by late vascular changes. Diabetologia.

[18] Yang Q., Xu Y., Xie P., Cheng H., Song Q., Su T., Yuan S., Liu Q. (2015). Retinal Neurodegeneration in db/db Mice at the Early Period of Diabetes. J. Ophthalmol..

[19] Abu-El-Asrar A.M., Dralands L., Missotten L., Al-Jadaan I.A., Geboes K. (2004). Expression of apoptosis markers in the retinas of human subjects with diabetes. Investig. Ophthalmol. Vis. Sci..

[20] Nadal-Nicolas F.M., Jimenez-Lopez M., Sobrado-Calvo P., Nieto-Lopez L., Canovas-Martinez I., Salinas-Navarro M., Vidal-Sanz M., Agudo M. (2009). Brn3a as a marker of retinal ganglion cells: Qualitative and quantitative time course studies in naive and optic nerve-injured retinas. Investig. Ophthalmol. Vis. Sci..

[21] Yang Y., Mao D., Chen X., Zhao L., Tian Q., Liu C., Zhou B.L. (2012). Decrease in retinal neuronal cells in streptozotocin-induced diabetic mice. Mol. Vis..

[22] Fernandez D.C., Sande P.H., de Zavalia N., Belforte N., Dorfman D., Casiraghi L.P., Golombek D., Rosenstein R.E. (2013). Effect of experimental diabetic retinopathy on the non-image-forming visual system. Chronobiol. Int..

[23] Hombrebueno J.R., Chen M., Penalva R.G., Xu H. (2014). Loss of synaptic connectivity, particularly in second order neurons is a key feature of diabetic retinal neuropathy in the Ins2Akita mouse. PLoS ONE.

[24] Jiang H., Du J., Song J., Li Y., Wu M., Zhou J., Wu S. (2018). Loss-of-function mutation of serine racemase attenuates retinal ganglion cell loss in diabetic mice. Exp. Eye Res..

[25] Ma M., Xu Y., Xiong S., Zhang J., Gu Q., Ke B., Xu X. (2018). Involvement of ciliary neurotrophic factor in early diabetic retinal neuropathy in streptozotocin-induced diabetic rats. Eye.

[26] Church K.A., Rodriguez D., Vanegas D., Gutierrez I.L., Cardona S.M., Madrigal J.L.M., Kaur T., Cardona A.E. (2022). Models of microglia depletion and replenishment elicit protective effects to alleviate vascular and neuronal damage in the diabetic murine retina. J. Neuroinflamm..

[27] Piri N., Kwong J.M., Song M., Caprioli J. (2006). Expression of hermes gene is restricted to the ganglion cells in the retina. Neurosci. Lett..

[28] Kowluru R.A., Abbas S.N. (2003). Diabetes-induced mitochondrial dysfunction in the retina. Investig. Ophthalmol. Vis. Sci..

[29] Adamiec-Mroczek J., Zajac-Pytrus H., Misiuk-Hojlo M. (2015). Caspase-Dependent Apoptosis of Retinal Ganglion Cells During the Development of Diabetic Retinopathy. Adv. Clin. Exp. Med..

[30] Park H.L., Kim J.H., Park C.K. (2018). Different contributions of autophagy to retinal ganglion cell death in the diabetic and glaucomatous retinas. Sci. Rep..

[31] Giardino I., Fard A.K., Hatchell D.L., Brownlee M. (1998). Aminoguanidine inhibits reactive oxygen species formation, lipid peroxidation, and oxidant-induced apoptosis. Diabetes.

[32] Baynes J.W., Thorpe S.R. (1999). Role of oxidative stress in diabetic complications: A new perspective on an old paradigm. Diabetes.

[33] Alka K., Kumar J., Kowluru R.A. (2023). Impaired mitochondrial dynamics and removal of the damaged mitochondria in diabetic retinopathy. Front. Endocrinol..

[34] Malaviya P., Kowluru R.A. (2024). Homocysteine and mitochondrial quality control in diabetic retinopathy. Eye Vision.

[35] Malaviya P., Kumar J., Kowluru R.A. (2024). Role of ferroptosis in mitochondrial damage in diabetic retinopathy. Free Radic. Biol. Med..

[36] Zhao M.H., Hu J., Li S., Wu Q., Lu P. (2018). P66Shc expression in diabetic rat retina. BMC Ophthalmol..

[37] Mishra M., Duraisamy A.J., Bhattacharjee S., Kowluru R.A. (2019). Adaptor Protein p66Shc: A Link Between Cytosolic and Mitochondrial Dysfunction in the Development of Diabetic Retinopathy. Antioxid. Redox Signal..

[38] Zhu H., Zhang W., Zhao Y., Shu X., Wang W., Wang D., Yang Y., He Z., Wang X., Ying Y. (2018). GSK3beta-mediated tau hyperphosphorylation triggers diabetic retinal neurodegeneration by disrupting synaptic and mitochondrial functions. Mol. Neurodegener..

[39] Haider S.Z., Sadanandan N.P., Joshi P.G., Mehta B. (2019). Early Diabetes Induces Changes in Mitochondrial Physiology of Inner Retinal Neurons. Neuroscience.

[40] Santiago A.R., Rosa S.C., Santos P.F., Cristovao A.J., Barber A.J., Ambrosio A.F. (2006). Elevated glucose changes the expression of ionotropic glutamate receptor subunits and impairs calcium homeostasis in retinal neural cells. Investig. Ophthalmol. Vis. Sci..

[41] Alam N.M., Mills W.C.t., Wong A.A., Douglas R.M., Szeto H.H., Prusky G.T. (2015). A mitochondrial therapeutic reverses visual decline in mouse models of diabetes. Dis. Models Mech..

[42] Daniel A., Premilovac D., Foa L., Feng Z., Shah K., Zhang Q., Woolley K.L., Bye N., Smith J.A., Gueven N. (2021). Novel Short-Chain Quinones to Treat Vision Loss in a Rat Model of Diabetic Retinopathy. Int. J. Mol. Sci..

[43] Lam C.H., Zuo B., Chan H.H., Leung T.W., Abokyi S., Catral K.P.C., Tse D.Y. (2024). Coenzyme Q10 eyedrops conjugated with vitamin E TPGS alleviate neurodegeneration and mitochondrial dysfunction in the diabetic mouse retina. Front. Cell. Neurosci..

[44] Xu Z., Wei Y., Gong J., Cho H., Park J.K., Sung E.R., Huang H., Wu L., Eberhart C., Handa J.T. (2014). NRF2 plays a protective role in diabetic retinopathy in mice. Diabetologia.

[45] Kowluru R.A., Mishra M. (2017). Epigenetic regulation of redox signaling in diabetic retinopathy: Role of Nrf2. Free Radic. Biol. Med..

[46] Mishra M., Zhong Q., Kowluru R.A. (2014). Epigenetic modifications of Nrf2-mediated glutamate-cysteine ligase: Implications for the development of diabetic retinopathy and the metabolic memory phenomenon associated with its continued progression. Free Radic. Biol. Med..

[47] Mishra M., Zhong Q., Kowluru R.A. (2014). Epigenetic modifications of Keap1 regulate its interaction with the protective factor Nrf2 in the development of diabetic retinopathy. Investig. Ophthalmol. Vis. Sci..

[48] Fang J., Bai W., Yang L. (2023). Astaxanthin inhibits oxidative stress and apoptosis in diabetic retinopathy. Acta Histochem..

[49] Yeh P.T., Huang H.W., Yang C.M., Yang W.S., Yang C.H. (2016). Astaxanthin Inhibits Expression of Retinal Oxidative Stress and Inflammatory Mediators in Streptozotocin-Induced Diabetic Rats. PLoS ONE.

[50] Dong L.Y., Jin J., Lu G., Kang X.L. (2013). Astaxanthin attenuates the apoptosis of retinal ganglion cells in db/db mice by inhibition of oxidative stress. Mar. Drugs.

[51] Chous A.P., Richer S.P., Gerson J.D., Kowluru R.A. (2016). The Diabetes Visual Function Supplement Study (DiVFuSS). Br. J. Ophthalmol..

[52] Baccouche B., Benlarbi M., Barber A.J., Ben Chaouacha-Chekir R. (2018). Short-Term Administration of Astaxanthin Attenuates Retinal Changes in Diet-Induced Diabetic *Psammomys obesus*. Curr. Eye Res..

[53] Kim S.J., Yoo W.S., Choi M., Chung I., Yoo J.M., Choi W.S. (2016). Increased O-GlcNAcylation of NF-kappaB Enhances Retinal Ganglion Cell Death in Streptozotocin-induced Diabetic Retinopathy. Curr. Eye Res..

[54] Miller W.P., Mihailescu M.L., Yang C., Barber A.J., Kimball S.R., Jefferson L.S., Dennis M.D. (2016). The Translational Repressor 4E-BP1 Contributes to Diabetes-Induced Visual Dysfunction. Investig. Ophthalmol. Vis. Sci..

[55] Miller W.P., Yang C., Mihailescu M.L., Moore J.A., Dai W., Barber A.J., Dennis M.D. (2018). Deletion of the Akt/mTORC1 Repressor REDD1 Prevents Visual Dysfunction in a Rodent Model of Type 1 Diabetes. Diabetes.

[56] Dierschke S.K., Miller W.P., Favate J.S., Shah P., Imamura Kawasawa Y., Salzberg A.C., Kimball S.R., Jefferson L.S., Dennis M.D. (2019). O-GlcNAcylation alters the selection of mRNAs for translation and promotes 4E-BP1-dependent mitochondrial dysfunction in the retina. J. Biol. Chem..

[57] Miller W.P., Toro A.L., Barber A.J., Dennis M.D. (2019). REDD1 Activates a ROS-Generating Feedback Loop in the Retina of Diabetic Mice. Investig. Ophthalmol. Vis. Sci..

[58] Xu Y., Ola M.S., Berkich D.A., Gardner T.W., Barber A.J., Palmieri F., Hutson S.M., Lanoue K.F. (2007). Energy sources for glutamate neurotransmission in the retina: Absence of the aspartate/glutamate carrier produces reliance on glycolysis in glia. J. Neurochem..

[59] Barber A.J., Robinson W.F., Jackson G.R., Tombran-Tink J., Barnstable C.J., Gardner T.W. (2012). Neurodegeneration in Diabetic Retinopathy. Visual Dysfunction in Diabetes: The Science of Patient Impairment and Health Care.

[60] Barber A.J., Gardner T.W., Abcouwer S.F. (2011). The significance of vascular and neural apoptosis to the pathology of diabetic retinopathy. Investig. Ophthalmol. Vis. Sci..

[61] Lieth E., LaNoue K.F., Antonetti D.A., Ratz M. (2000). Diabetes reduces glutamate oxidation and glutamine synthesis in the retina. Exp. Eye Res..

[62] Kowluru R.A., Engerman R.L., Case G.L., Kern T.S. (2001). Retinal glutamate in diabetes and effect of antioxidants. Neurochem. Int..

[63] Lieth E., LaNoue K.F., Berkich D.A., Xu B., Ratz M., Taylor C., Hutson S.M. (2001). Nitrogen shuttling between neurons and glial cells during glutamate synthesis. J. Neurochem..

[64] Hutson S.M., Lieth E., LaNoue K.F. (2001). Function of leucine in excitatory neurotransmitter metabolism in the central nervous system. J. Nutr..

[65] Gowda K., Zinnanti W.J., LaNoue K.F. (2011). The influence of diabetes on glutamate metabolism in retinas. J. Neurochem..

[66] Zhang X., Xia M., Wu Y., Zhang F. (2023). Branched-Chain Amino Acids Metabolism and Their Roles in Retinopathy: From Relevance to Mechanism. Nutrients.

[67] Lau J.C., Kroes R.A., Moskal J.R., Linsenmeier R.A. (2013). Diabetes changes expression of genes related to glutamate neurotransmission and transport in the Long-Evans rat retina. Mol. Vis..

[68] Chihara E., Matsuoka T., Ogura Y., Matsumura M. (1993). Retinal nerve fiber layer defect as an early manifestation of diabetic retinopathy. Ophthalmology.

[69] Ozdek S., Lonneville Y.H., Onol M., Yetkin I., Hasanreisoglu B.B. (2002). Assessment of nerve fiber layer in diabetic patients with scanning laser polarimetry. Eye.

[70] Biallosterski C., van Velthoven M.E., Michels R.P., Schlingemann R.O., DeVries J.H., Verbraak F.D. (2007). Decreased optical coherence tomography-measured pericentral retinal thickness in patients with diabetes mellitus type 1 with minimal diabetic retinopathy. Br. J. Ophthalmol..

[71] Cabrera DeBuc D., Somfai G.M. (2010). Early detection of retinal thickness changes in diabetes using Optical Coherence Tomography. Med. Sci. Monit..

[72] van Dijk H.W., Kok P.H.B., Garvin M., Sonka M., Devries J.H., Michels R.P.J., van Velthoven M.E.J., Schlingemann R.O., Verbraak F.D., Abramoff M.D. (2009). Selective loss of inner retinal layer thickness in type 1 diabetic patients with minimal diabetic retinopathy. Investig. Ophthalmol. Vis. Sci..

[73] van Dijk H.W., Verbraak F.D., Kok P.H., Garvin M.K., Sonka M., Lee K., Devries J.H., Michels R.P., van Velthoven M.E., Schlingemann R.O. (2010). Decreased retinal ganglion cell layer thickness in patients with type 1 diabetes. Investig. Ophthalmol. Vis. Sci..

[74] van Dijk H.W., Verbraak F.D., Kok P.H., Stehouwer M., Garvin M.K., Sonka M., DeVries J.H., Schlingemann R.O., Abramoff M.D. (2012). Early neurodegeneration in the retina of type 2 diabetic patients. Investig. Ophthalmol. Vis. Sci..

[75] Hammoum I., Benlarbi M., Dellaa A., Szabo K., Dekany B., Csaba D., Almasi Z., Hajdu R.I., Azaiz R., Charfeddine R. (2017). Study of retinal neurodegeneration and maculopathy in diabetic Meriones shawi: A particular animal model with human-like macula. J. Comp. Neurol..

[76] Lam C.H., Zou B., Chan H.H., Tse D.Y. (2023). Functional and structural changes in the neuroretina are accompanied by mitochondrial dysfunction in a type 2 diabetic mouse model. Eye Vision.

[77] van Dijk H.W., Verbraak F.D., Stehouwer M., Kok P.H., Garvin M.K., Sonka M., DeVries J.H., Schlingemann R.O., Abramoff M.D. (2011). Association of visual function and ganglion cell layer thickness in patients with diabetes mellitus type 1 and no or minimal diabetic retinopathy. Vision Res..

[78] Montesano G., Ometto G., Higgins B.E., Das R., Graham K.W., Chakravarthy U., McGuiness B., Young I.S., Kee F., Wright D.M. (2021). Evidence for Structural and Functional Damage of the Inner Retina in Diabetes With No Diabetic Retinopathy. Investig. Ophthalmol. Vis. Sci..

[79] Zhu T., Ma J., Li Y., Zhang Z. (2015). Association between retinal neuronal degeneration and visual function impairment in type 2 diabetic patients without diabetic retinopathy. Sci. China Life Sci..

[80] Kim J.H., Lee M.W., Byeon S.H., Kim S.S., Koh H.J., Lee S.C., Kim M. (2018). Associations between Individual Retinal Layer Thicknesses and Diabetic Peripheral Neuropathy Using Retinal Layer Segmentation Analysis. Retina.

[81] Chen Y., Li J., Yan Y., Shen X. (2016). Diabetic macular morphology changes may occur in the early stage of diabetes. BMC Ophthalmol..

[82] De Clerck E.E., Schouten J.S., Berendschot T.T., Kessels A.G., Nuijts R.M., Beckers H.J., Schram M.T., Stehouwer C.D., Webers C.A. (2015). New ophthalmologic imaging techniques for detection and monitoring of neurodegenerative changes in diabetes: A systematic review. Lancet Diabetes Endocrinol..

[83] De Clerck E.E., Schouten J.S., Berendschot T.T., Beckers H.J., Schaper N.C., Schram M.T., Stehouwer C.D., Webers C.A. (2017). Loss of Temporal Peripapillary Retinal Nerve Fibers in Prediabetes or Type 2 Diabetes Without Diabetic Retinopathy: The Maastricht StudyTemporal RNFL Thinning in Prediabetes. Investig. Ophthalmol. Vis. Sci..

[84] Sohn E.H., van Dijk H.W., Jiao C., Kok P.H., Jeong W., Demirkaya N., Garmager A., Wit F., Kucukevcilioglu M., van Velthoven M.E. (2016). Retinal neurodegeneration may precede microvascular changes characteristic of diabetic retinopathy in diabetes mellitus. Proc. Natl. Acad. Sci. USA.

[85] Lee M.W., Lim H.B., Kim M.S., Park G.S., Nam K.Y., Lee Y.H., Kim J.Y. (2021). Effects of prolonged type 2 diabetes on changes in peripapillary retinal nerve fiber layer thickness in diabetic eyes without clinical diabetic retinopathy. Sci. Rep..

[86] Moghimi S., Zangwill L.M., Penteado R.C., Hasenstab K., Ghahari E., Hou H., Christopher M., Yarmohammadi A., Manalastas P.I.C., Shoji T. (2018). Macular and Optic Nerve Head Vessel Density and Progressive Retinal Nerve Fiber Layer Loss in Glaucoma. Ophthalmology.

[87] Yu M., Lin C., Weinreb R.N., Lai G., Chiu V., Leung C.K. (2016). Risk of Visual Field Progression in Glaucoma Patients with Progressive Retinal Nerve Fiber Layer Thinning: A 5-Year Prospective Study. Ophthalmology.

[88] Miki A., Medeiros F.A., Weinreb R.N., Jain S., He F., Sharpsten L., Khachatryan N., Hammel N., Liebmann J.M., Girkin C.A. (2014). Rates of retinal nerve fiber layer thinning in glaucoma suspect eyes. Ophthalmology.

[89] Sung K.R., Sun J.H., Na J.H., Lee J.Y., Lee Y. (2012). Progression detection capability of macular thickness in advanced glaucomatous eyes. Ophthalmology.

[90] Wall K., Arend L.P., von der Emde L., Sasmannshausen M., Holz F.G., Ach T. (2025). Characterization of the Disorganization of the Inner Retinal Layers in Diabetics Using Increased Axial Resolution Optical Coherence Tomography. TVST.

[91] Chung C.Y., Koprich J.B., Siddiqi H., Isacson O. (2009). Dynamic changes in presynaptic and axonal transport proteins combined with striatal neuroinflammation precede dopaminergic neuronal loss in a rat model of AAV alpha-synucleinopathy. J. Neurosci..

[92] Anderson E.E., Greferath U., Fletcher E.L. (2016). Changes in morphology of retinal ganglion cells with eccentricity in retinal degeneration. Cell Tissue Res..

[93] Kern T.S., Barber A.J. (2008). Retinal ganglion cells in diabetes. J. Physiol..

[94] Meyer-Rusenberg B., Pavlidis M., Stupp T., Thanos S., Meyer-Rusenberg B., Pavlidis M., Stupp T., Thanos S. (2007). Pathological changes in human retinal ganglion cells associated with diabetic and hypertensive retinopathy. Graefe’s Arch. Clin. Exp. Ophthalmol..

[95] Son J.R., Lee M.J., Jeon C.J. (2023). Changes in Starburst Amacrine Cells in Mice with Diabetic Retinopathy. Front. Biosci..

[96] Ivanova E., Bianchimano P., Corona C., Eleftheriou C.G., Sagdullaev B.T. (2020). Optogenetic Stimulation of Cholinergic Amacrine Cells Improves Capillary Blood Flow in Diabetic Retinopathy. Investig. Ophthalmol. Vis. Sci..

[97] Enzsoly A., Szabo A., Szabo K., Szel A., Nemeth J., Lukats A. (2015). Novel features of neurodegeneration in the inner retina of early diabetic rats. Histol. Histopathol..

[98] VanGuilder H.D., Brucklacher R.M., Patel K., Ellis R.W., Freeman W.M., Barber A.J. (2008). Diabetes downregulates presynaptic proteins and reduces basal synapsin I phosphorylation in rat retina. Eur. J. Neurosci..

[99] D’Cruz T.S., Weibley B.N., Kimball S.R., Barber A.J. (2012). Post-translational processing of synaptophysin in the rat retina is disrupted by diabetes. PLoS ONE.

[100] Gaspar J.M., Baptista F.I., Galvao J., Castilho A.F., Cunha R.A., Ambrosio A.F. (2010). Diabetes differentially affects the content of exocytotic proteins in hippocampal and retinal nerve terminals. Neuroscience.

[101] Sundstrom J.M., Hernandez C., Weber S.R., Zhao Y., Dunklebarger M., Tiberti N., Laremore T., Simo-Servat O., Garcia-Ramirez M., Barber A.J. (2018). Proteomic Analysis of Early Diabetic Retinopathy Reveals Mediators of Neurodegenerative Brain Diseases. Investig. Ophthalmol. Vis. Sci..

[102] Shankar U., Gunasundari R. (2015). A Review on Electrophysiology Based Detection of Diabetic Retinopathy. Procedia Comput. Sci..

[103] Bui B.V., Fortune B. (2004). Ganglion cell contributions to the rat full-field electroretinogram. J. Physiol..

[104] Kohzaki K., Vingrys A.J., Bui B.V., Kohzaki K., Vingrys A.J., Bui B.V. (2008). Early inner retinal dysfunction in streptozotocin-induced diabetic rats. Investig. Ophthalmol. Vis. Sci..

[105] Bui B.V., Loeliger M., Thomas M., Vingrys A.J., Rees S.M., Nguyen C.T.O., He Z., Tolcos M. (2009). Investigating structural and biochemical correlates of ganglion cell dysfunction in streptozotocin-induced diabetic rats. Exp. Eye Res..

[106] Ha Y., Saul A., Tawfik A., Zorrilla E.P., Ganapathy V., Smith S.B. (2012). Diabetes accelerates retinal ganglion cell dysfunction in mice lacking sigma receptor 1. Mol. Vis..

[107] Nasralah Z., Robinson W., Jackson G., Barber A. (2013). Measuring Visual Function in Diabetic Retinopathy: Progress in Basic and Clinical Research. Clin. Exp. Ophthalmol..

[108] Castoldi V., Zerbini G., Maestroni S., Vigano I., Rama P., Leocani L. (2023). Topical Nerve Growth Factor (NGF) restores electrophysiological alterations in the Ins2Akita mouse model of diabetic retinopathy. Exp. Eye Res..

[109] Abraham F.A., Haimovitz J., Berezin M. (1988). The photopic and scotopic visual thresholds in diabetics without diabetic retinopathy. Metab. Pediatr. Syst. Ophthalmol..

[110] Aylward G.W., Billson F.A. (1989). The scotopic threshold response in diabetic retinopathy—A preliminary report. Aust. N. Z. J. Ophthalmol..

[111] Parisi V., Uccioli L. (2001). Visual electrophysiological responses in persons with type 1 diabetes. Diabetes Metab. Res. Rev..

[112] Berardi N., Domenici L., Gravina A., Maffei L. (1990). Pattern ERG in rats following section of the optic nerve. Exp. Brain Res..

[113] Ding Y., Yuan S., Liu X., Mao P., Zhao C., Huang Q., Zhang R., Fang Y., Song Q., Yuan D. (2014). Protective effects of astragaloside IV on db/db mice with diabetic retinopathy. PLoS ONE.

[114] Yuan D., Xu Y., Hang H., Liu X., Chen X., Xie P., Yuan S., Zhang W., Lin X., Liu Q. (2014). Edaravone protect against retinal damage in streptozotocin-induced diabetic mice. PLoS ONE.

[115] Dionysopoulou S., Wikstrom P., Bucolo C., Romano G.L., Micale V., Svensson R., Spyridakos D., Mastrodimou N., Georgakis S., Verginis P. (2023). Topically Administered NOX4 Inhibitor, GLX7013114, Is Efficacious in Treating the Early Pathological Events of Diabetic Retinopathy. Diabetes.

[116] Boschi M.C., Frosini R., Mencucci R., Sodi A. (1989). The influence of early diabetes on the pattern electroretinogram. Doc. Ophthalmol..

[117] Falsini B., Porciatti V., Scalia G., Caputo S., Minnella A., Di Leo M.A., Ghirlanda G. (1989). Steady-state pattern electroretinogram in insulin-dependent diabetics with no or minimal retinopathy. Doc. Ophthalmol..

[118] Ghirlanda G., Di Leo M.A., Caputo S., Falsini B., Porciatti V., Marietti G., Greco A.V. (1991). Detection of inner retina dysfunction by steady-state focal electroretinogram pattern and flicker in early IDDM. Diabetes.

[119] Parisi V., Uccioli L., Monticone G., Parisi L., Manni G., Ippoliti D., Menzinger G., Bucci M.G. (1997). Electrophysiological assessment of visual function in IDDM patients. Electroencephalogr. Clin. Neurophysiol..

[120] Prager T.C., Garcia C.A., Mincher C.A., Mishra J., Chu H.H. (1990). The pattern electroretinogram in diabetes. Am. J. Ophthalmol..

[121] Di Leo M.A., Falsini B., Caputo S., Ghirlanda G., Porciatti V., Greco A.V. (1990). Spatial frequency-selective losses with pattern electroretinogram in type 1 (insulin-dependent) diabetic patients without retinopathy. Diabetologia.

[122] Caputo S., Di Leo M.A., Falsini B., Ghirlanda G., Porciatti V., Minella A., Greco A.V. (1990). Evidence for early impairment of macular function with pattern ERG in type I diabetic patients. Diabetes Care.

[123] Deak K., Fejes I., Janaky M., Varkonyi T., Benedek G., Braunitzer G. (2016). Further Evidence for the Utility of Electrophysiological Methods for the Detection of Subclinical Stage Retinal and Optic Nerve Involvement in Diabetes. Med. Princ. Pract..

[124] Mermeklieva E.A. (2019). Pattern electroretinography and retinal changes in patients with diabetes mellitus type 2. Neurophysiol. Clin..

[125] Lasta M., Pemp B., Schmidl D., Boltz A., Kaya S., Palkovits S., Werkmeister R., Howorka K., Popa-Cherecheanu A., Garhofer G. (2013). Neurovascular dysfunction precedes neural dysfunction in the retina of patients with type 1 diabetes. Investig. Ophthalmol. Vis. Sci..

[126] Lecleire-Collet A., Audo I., Aout M., Girmens J.F., Sofroni R., Erginay A., Le Gargasson J.F., Mohand-Said S., Meas T., Guillausseau P.J. (2011). Evaluation of retinal function and flicker light-induced retinal vascular response in normotensive patients with diabetes without retinopathy. Investig. Ophthalmol. Vis. Sci..

[127] Kocer A.M., Sekeroglu M.A. (2021). Evaluation of the neuronal and microvascular components of the macula in patients with diabetic retinopathy. Doc. Ophthalmol..

[128] Polat Gultekin B., Hamurcu M. (2024). Evaluation of optical coherence tomography angiography and pattern and flash electroretinography in diabetes mellitus without retinopathy. Ann. Med..

[129] Kuznetsov K.I., Veselovskaya N.N., Maslov V.Y., Fedulova S.A., Veselovsky M.S. (2014). Electrical Properties of Retinal Ganglion Cells of the Rats with Streptozotocin-Induced Diabetes Mellitus. Int. J. Phys. Pathophysiol..

[130] Martyniuk N.Y., Maslov V.Y., Purnyn H.E., Fedulova S.A., Veselovsky N.S. (2018). Changes in Ongoing Activity and Electrophysiological Characteristics of the Rat Retinal Ganglion Cells in Diabetes Mellitus. Int. J. Phys. Pathophysiol..

[131] Yu J., Wang L., Weng S.J., Yang X.L., Zhang D.Q., Zhong Y.M. (2013). Hyperactivity of ON-type retinal ganglion cells in streptozotocin-induced diabetic mice. PLoS ONE.

[132] Moore-Dotson J.M., Eggers E.D. (2019). Reductions in Calcium Signaling Limit Inhibition to Diabetic Retinal Rod Bipolar Cells. Investig. Ophthalmol. Vis. Sci..

[133] Pu M., Xu L., Zhang H. (2006). Visual response properties of retinal ganglion cells in the royal college of surgeons dystrophic rat. Investig. Ophthalmol. Vis. Sci..

[134] Xiao C., He M., Nan Y., Zhang D., Chen B., Guan Y., Pu M. (2012). Physiological effects of superoxide dismutase on altered visual function of retinal ganglion cells in db/db mice. PLoS ONE.

[135] Zhou Y., Xiao C., Pu M. (2017). High glucose levels impact visual response properties of retinal ganglion cells in C57 mice-An in vitro physiological study. Sci. China Life Sci..

[136] Cui R.Z., Wang L., Qiao S.N., Wang Y.C., Wang X., Yuan F., Weng S.J., Yang X.L., Zhong Y.M. (2019). ON-Type Retinal Ganglion Cells are Preferentially Affected in STZ-Induced Diabetic Mice. Investig. Ophthalmol. Vis. Sci..

[137] Wang Q., So C., Qiu C., Zhang T., Yang K., Pan F. (2024). Diminished light sensitivities of ON alpha retinal ganglion cells observed in a mouse model of hyperglycemia. Exp. Eye Res..

[138] Trenholm S., Awatramani G.B. (2015). Origins of spontaneous activity in the degenerating retina. Front. Cell. Neurosci..

[139] Flood M.D., Wellington A.J., Cruz L.A., Eggers E.D. (2020). Early diabetes impairs ON sustained ganglion cell light responses and adaptation without cell death or dopamine insensitivity. Exp. Eye Res..

[140] Flood M.D., Wellington A.J., Eggers E.D. (2022). Impaired Light Adaptation of ON-Sustained Ganglion Cells in Early Diabetes Is Attributable to Diminished Response to Dopamine D4 Receptor Activation. Investig. Ophthalmol. Vis. Sci..

[141] MacIsaac A.R., Wellington A.J., Filicetti K., Eggers E.D. (2024). Impaired dopamine signaling in early diabetic retina: Insights from D1R and D4R agonist effects on whole retina responses. Exp. Eye Res..

[142] Motz C.T., Chesler K.C., Allen R.S., Bales K.L., Mees L.M., Feola A.J., Maa A.Y., Olson D.E., Thule P.M., Iuvone P.M. (2020). Novel Detection and Restorative Levodopa Treatment for Preclinical Diabetic Retinopathy. Diabetes.

[143] Aung M.H., Park H.N., Han M.K., Obertone T.S., Abey J., Aseem F., Thule P.M., Iuvone P.M., Pardue M.T. (2014). Dopamine deficiency contributes to early visual dysfunction in a rodent model of type 1 diabetes. J. Neurosci..

[144] Schiller P.H., Sandell J.H., Maunsell J.H. (1986). Functions of the ON and OFF channels of the visual system. Nature.

[145] Schiller P.H. (1992). The ON and OFF channels of the visual system. Trends Neurosci..

[146] Moore-Dotson J.M., Beckman J.J., Mazade R.E., Hoon M., Bernstein A.S., Romero-Aleshire M.J., Brooks H.L., Eggers E.D. (2016). Early Retinal Neuronal Dysfunction in Diabetic Mice: Reduced Light-Evoked Inhibition Increases Rod Pathway Signaling. Investig. Ophthalmol. Vis. Sci..

[147] Castilho A., Ambrosio A.F., Hartveit E., Veruki M.L. (2015). Disruption of a neural microcircuit in the rod pathway of the mammalian retina by diabetes mellitus. J. Neurosci..

[148] Castilho A., Madsen E., Ambrosio A.F., Veruki M.L., Hartveit E. (2015). Diabetic hyperglycemia reduces Ca2+ permeability of extrasynaptic AMPA receptors in AII amacrine cells. J. Neurophysiol..

[149] Patterson S.S., Cai Y., Yang Q., Merigan W.H., Williams D.R. (2024). Asymmetric Activation of Retinal ON and OFF Pathways by AOSLO Raster-Scanned Visual Stimuli. bioRxiv.

[150] Freed M.A. (2017). Asymmetry between ON and OFF α ganglion cells of mouse retina: Integration of signal and noise from synaptic inputs. J. Physiol..

[151] Eggers E.D., Carreon T.A. (2020). The effects of early diabetes on inner retinal neurons. Vis. Neurosci..

[152] Feng L., Zhao Y., Yoshida M., Chen H., Yang J.F., Kim T.S., Cang J., Troy J.B., Liu X. (2013). Sustained ocular hypertension induces dendritic degeneration of mouse retinal ganglion cells that depends on cell type and location. Investig. Ophthalmol. Vis. Sci..

[153] Li R.S., Chen B.Y., Tay D.K., Chan H.H., Pu M.L., So K.F. (2006). Melanopsin-expressing retinal ganglion cells are more injury-resistant in a chronic ocular hypertension model. Investig. Ophthalmol. Vis. Sci..

[154] LaVail J.H., Tauscher A.N., Aghaian E., Harrabi O., Sidhu S.S. (2003). Axonal transport and sorting of herpes simplex virus components in a mature mouse visual system. J. Virol..

[155] Fahy E.T., Chrysostomou V., Crowston J.G. (2016). Mini-Review: Impaired Axonal Transport and Glaucoma. Curr. Eye Res..

[156] Scott T.M., Foote J., Peat B., Galway G. (1986). Vascular and neural changes in the rat optic nerve following induction of diabetes with streptozotocin. J. Anat..

[157] Howell S.J., Mekhail M.N., Azem R., Ward N.L., Kern T.S. (2013). Degeneration of retinal ganglion cells in diabetic dogs and mice: Relationship to glycemic control and retinal capillary degeneration. Mol. Vis..

[158] Li S., Wang X., Yang J., Lei H., Wang X., Xiang Y. (2017). Metabolic profile of visual cortex in diabetic rats measured with in vivo proton MRS. NMR Biomed..

[159] Medori R., Autilio-Gambetti L., Monaco S., Gambetti P. (1985). Experimental diabetic neuropathy: Impairment of slow transport with changes in axon cross-sectional area. Proc. Natl. Acad. Sci. USA.

[160] Tsukada T., Chihara E. (1986). Changes in components of fast axonally transported proteins in the optic nerves of diabetic rabbits. Investig. Ophthalmol. Vis. Sci..

[161] Zhang L., Ino-ue M., Dong K., Yamamoto M. (2000). Retrograde axonal transport impairment of large- and medium-sized retinal ganglion cells in diabetic rat. Curr. Eye Res..

[162] Ino-Ue M., Zhang L., Naka H., Kuriyama H., Yamamoto M. (2000). Polyol metabolism of retrograde axonal transport in diabetic rat large optic nerve fiber. Investig. Ophthalmol. Vis. Sci..

[163] Fernandez D.C., Pasquini L.A., Dorfman D., Aldana Marcos H.J., Rosenstein R.E. (2012). Ischemic conditioning protects from axoglial alterations of the optic pathway induced by experimental diabetes in rats. PLoS ONE.

[164] Dorfman D., Aranda M.L., Rosenstein R.E. (2015). Enriched Environment Protects the Optic Nerve from Early Diabetes-Induced Damage in Adult Rats. PLoS ONE.

[165] Foxton R., Osborne A., Martin K.R., Ng Y.S., Shima D.T. (2016). Distal retinal ganglion cell axon transport loss and activation of p38 MAPK stress pathway following VEGF-A antagonism. Cell Death Dis..

[166] Delcroix J.D., Tomlinson D.R., Fernyhough P. (1997). Diabetes and axotomy-induced deficits in retrograde axonal transport of nerve growth factor correlate with decreased levels of p75LNTR protein in lumbar dorsal root ganglia. Brain Res. Mol. Brain Res..

[167] Hellweg R., Raivich G., Hartung H.D., Hock C., Kreutzberg G.W. (1994). Axonal transport of endogenous nerve growth factor (NGF) and NGF receptor in experimental diabetic neuropathy. Exp. Neurol..

[168] Fernyhough P., Diemel L.T., Hardy J., Brewster W.J., Mohiuddin L., Tomlinson D.R. (1995). Human recombinant nerve growth factor replaces deficient neurotrophic support in the diabetic rat. Eur. J. Neurosci..

[169] Juranek J.K., Geddis M.S., Rosario R., Schmidt A.M. (2013). Impaired slow axonal transport in diabetic peripheral nerve is independent of RAGE. Eur. J. Neurosci..

[170] Zhao J.P., Ma Z.Z., Song C., Li X.H., Li Y.Z., Liu Y.Y. (2010). Optic nerve lesions in diabetic rats: Blood flow to the optic nerve, permeability of micro blood vessels and histopathology. Int. J. Ophthalmol..

[171] Sredy J., Sawicki D.R., Notvest R.R. (1991). Polyol pathway activity in nervous tissues of diabetic and galactose-fed rats: Effect of dietary galactose withdrawal or tolrestat intervention therapy. J. Diabet. Complicat..

[172] Mendonca H.R., Carvalho J.N.A., Abreu C.A., Mariano de Souza Aguiar Dos Santos D., Carvalho J.R., Marques S.A., da Costa Calaza K., Martinez A.M.B. (2018). Lack of Galectin-3 attenuates neuroinflammation and protects the retina and optic nerve of diabetic mice. Brain Res..

[173] Catanzaro O.L., Capponi J.A., Di Martino I., Labal E.S., Sirois P. (2017). Oxidative stress in the optic nerve and cortical visual area of steptozotocin-induced diabetic Wistar rats: Blockade with a selective bradykinin B_1_ receptor antagonist. Neuropeptides.

[174] Casselini C.M., Parson H.K., Frizzi K.E., Marquez A., Smith D.R., Guernsey L., Nemmani R., Tayarani A., Jolivalt C.G., Weaver J. (2024). A muscarinic receptor antagonist reverses multiple indices of diabetic peripheral neuropathy: Preclinical and clinical studies using oxybutynin. Acta Neuropathol..

[175] Hattar S., Liao H.W., Takao M., Berson D.M., Yau K.W. (2002). Melanopsin-containing retinal ganglion cells: Architecture, projections, and intrinsic photosensitivity. Science.

[176] Perez-Leon J.A., Warren E.J., Allen C.N., Robinson D.W., Brown R.L. (2006). Synaptic inputs to retinal ganglion cells that set the circadian clock. Eur. J. Neurosci..

[177] Sondereker K.B., Stabio M.E., Renna J.M. (2020). Crosstalk: The diversity of melanopsin ganglion cell types has begun to challenge the canonical divide between image-forming and non-image-forming vision. J. Comp. Neurol..

[178] Berson D.M., Dunn F.A., Takao M. (2002). Phototransduction by retinal ganglion cells that set the circadian clock. Science.

[179] Chen W.Y., Han X., Cui L.J., Yu C.X., Sheng W.L., Yu J., Yuan F., Zhong Y.M., Yang X.L., Weng S.J. (2021). Cell-Subtype-Specific Remodeling of Intrinsically Photosensitive Retinal Ganglion Cells in Streptozotocin-Induced Diabetic Mice. Diabetes.

[180] Lahouaoui H., Coutanson C., Cooper H.M., Bennis M., Dkhissi-Benyahya O. (2014). Clock genes and behavioral responses to light are altered in a mouse model of diabetic retinopathy. PLoS ONE.

[181] Lahouaoui H., Coutanson C., Cooper H.M., Bennis M., Dkhissi-Benyahya O. (2016). Diabetic retinopathy alters light-induced clock gene expression and dopamine levels in the mouse retina. Mol. Vis..

[182] Kumar S., Zhuo L. (2011). Quantitative analysis of pupillary light reflex by real-time autofluorescent imaging in a diabetic mouse model. Exp. Eye Res..

[183] Straub R.H., Zietz B., Palitzsch K.D., Scholmerich J. (1996). Impact of disease duration on cardiovascular and pupillary autonomic nervous function in IDDM and NIDDM patients. Diabetes Care.

[184] Karachaliou F., Karavanaki K., Greenwood R., Baum J.D. (1997). Consistency of pupillary abnormality in children and adolescents with diabetes. Diabet. Med..

[185] Feigl B., Zele A.J., Fader S.M., Howes A.N., Hughes C.E., Jones K.A., Jones R. (2012). The post-illumination pupil response of melanopsin-expressing intrinsically photosensitive retinal ganglion cells in diabetes. Acta Ophthalmol..

[186] Ortube M.C., Kiderman A., Eydelman Y., Yu F., Aguilar N., Nusinowitz S., Gorin M.B. (2013). Comparative regional pupillography as a noninvasive biosensor screening method for diabetic retinopathy. Investig. Ophthalmol. Vis. Sci..

[187] Ishibashi F., Kojima R., Taniguchi M., Kosaka A., Uetake H., Tavakoli M. (2017). The Preferential Impairment of Pupil Constriction Stimulated by Blue Light in Patients with Type 2 Diabetes without Autonomic Neuropathy. J. Diabetes Res..

[188] Bista Karki S., Coppell K.J., Mitchell L.V., Ogbuehi K.C. (2020). Dynamic Pupillometry in Type 2 Diabetes: Pupillary Autonomic Dysfunction and the Severity of Diabetic Retinopathy. Clin. Ophthalmol..

[189] Tan T.E., Finkelstein M.T., Tan G.S.W., Tan A.C.S., Chan C.M., Mathur R., Wong E.Y.M., Cheung C.M.G., Wong T.Y., Milea D. (2022). Retinal neural dysfunction in diabetes revealed with handheld chromatic pupillometry. Clin. Exp. Ophthalmol..

[190] Hall C.A., Chilcott R.P. (2018). Eyeing up the Future of the Pupillary Light Reflex in Neurodiagnostics. Diagnostics.

[191] Dumpala S., Zele A.J., Feigl B. (2019). Outer Retinal Structure and Function Deficits Contribute to Circadian Disruption in Patients With Type 2 Diabetes. Investig. Ophthalmol. Vis. Sci..

[192] Kim J., Kim C.S., Lee Y.M., Sohn E., Jo K., Kim J.S. (2015). Litsea japonica extract inhibits neuronal apoptosis and the accumulation of advanced glycation end products in the diabetic mouse retina. Mol. Med. Rep..

[193] Zhang C., Xu Y., Tan H.Y., Li S., Wang N., Zhang Y., Feng Y. (2018). Neuroprotective effect of He-Ying-Qing-Re formula on retinal ganglion cell in diabetic retinopathy. J. Ethnopharmacol..

[194] Nabavi S.F., Barber A.J., Spagnuolo C., Russo G.L., Daglia M., Nabavi S.M., Sobarzo-Sanchez E. (2016). Nrf2 as molecular target for polyphenols: A novel therapeutic strategy in diabetic retinopathy. Crit. Rev. Clin. Lab. Sci..

[195] Nabavi S.F., Habtemariam S., Daglia M., Shafighi N., Barber A.J. (2015). Nabavi SM. Anthocyanins as a potential therapy for diabetic retinopathy. Curr. Med. Chem..

[196] Robledinos-Anton N., Fernandez-Gines R., Manda G., Cuadrado A. (2019). Activators and Inhibitors of NRF2: A Review of Their Potential for Clinical Development. Oxidative Med. Cell. Longev..

[197] Chen L., Qi E., Liu X., Cui L., Fan X., Wei T., Hu Y. (2024). The lack of homology domain and leucine rich repeat protein phosphatase 2 ameliorates visual impairment in rats with diabetic retinopathy through regulation of the AKT-GSK-3β-Nrf2 signal cascade. Toxicol. Appl. Pharmacol..

[198] Barber A.J., Baccouche B. (2017). Neurodegeneration in diabetic retinopathy: Potential for novel therapies. Vision Res..

[199] Kowluru R.A., Zhong Q., Santos J.M., Thandampallayam M., Putt D., Gierhart D.L. (2014). Beneficial effects of the nutritional supplements on the development of diabetic retinopathy. Nutr. Metab..

[200] Kowluru R.A., Menon B., Gierhart D.L. (2008). Beneficial effect of zeaxanthin on retinal metabolic abnormalities in diabetic rats. Investig. Ophthalmol. Vis. Sci..

[201] Yang F., Yu J., Ke F., Lan M., Li D., Tan K., Ling J., Wang Y., Wu K., Li D. (2018). Curcumin Alleviates Diabetic Retinopathy in Experimental Diabetic Rats. Ophthalmic Res..

[202] Yang X., Huo F., Liu B., Liu J., Chen T., Li J., Zhu Z., Lv B. (2017). Crocin Inhibits Oxidative Stress and Pro-inflammatory Response of Microglial Cells Associated with Diabetic Retinopathy Through the Activation of PI3K/Akt Signaling Pathway. J. Mol. Neurosci..

[203] Fan J., Xu G., Jiang T., Qin Y. (2012). Pharmacologic induction of heme oxygenase-1 plays a protective role in diabetic retinopathy in rats. Investig. Ophthalmol. Vis. Sci..

[204] Zhang Q., Guy K., Pagadala J., Jiang Y., Walker R.J., Liu L., Soderland C., Kern T.S., Ferry R., He H. (2012). Compound 49b prevents diabetes-induced apoptosis through increased IGFBP-3 levels. Investig. Ophthalmol. Vis. Sci..

[205] Foureaux G., Nogueira B.S., Coutinho D.C., Raizada M.K., Nogueira J.C., Ferreira A.J. (2015). Activation of endogenous angiotensin converting enzyme 2 prevents early injuries induced by hyperglycemia in rat retina. Braz. J. Med. Biol. Res..

[206] Hernandez C., Bogdanov P., Corraliza L., Garcia-Ramirez M., Sola-Adell C., Arranz J.A., Arroba A.I., Valverde A.M., Simo R. (2016). Topical Administration of GLP-1 Receptor Agonists Prevents Retinal Neurodegeneration in Experimental Diabetes. Diabetes.

[207] Liu Y., Leo L.F., McGregor C., Grivitishvili A., Barnstable C.J., Tombran-Tink J. (2012). Pigment epithelium-derived factor (PEDF) peptide eye drops reduce inflammation, cell death and vascular leakage in diabetic retinopathy in Ins2(Akita) mice. Mol. Med..

[208] Afarid M., Namvar E., Sanie-Jahromi F. (2020). Diabetic Retinopathy and BDNF: A Review on Its Molecular Basis and Clinical Applications. J. Ophthalmol..

[209] Le Y.Z., Xu B., Chucair-Elliott A.J., Zhang H., Zhu M. (2021). VEGF Mediates Retinal Muller Cell Viability and Neuroprotection through BDNF in Diabetes. Biomolecules.

[210] Reiter C.E., Sandirasegarane L., Wolpert E.B., Klinger M., Simpson I.A., Barber A.J., Antonetti D.A., Kester M., Gardner T.W. (2003). Characterization of insulin signaling in rat retina in vivo and ex vivo. Am. J. Physiol.—Endocrinol. Metab..

[211] Reiter C.E., Wu X., Sandirasegarane L., Nakamura M., Gilbert K.A., Singh R.S., Fort P.E., Antonetti D.A., Gardner T.W. (2006). Diabetes reduces basal retinal insulin receptor signaling: Reversal with systemic and local insulin. Diabetes.

[212] Barber A.J., Nakamura M., Wolpert E.B., Reiter C.E.N., Seigel G.M., Antonetti D.A., Gardner T.W. (2001). Insulin Rescues Retinal Neurons from Apoptosis by a Phosphatidylinositol 3-Kinase/Akt-mediated Mechanism That Reduces the Activation of Caspase-3. J. Biol. Chem..

[213] Faiq M.A., Sengupta T., Nath M., Velpandian T., Saluja D., Dada R., Dada T., Chan K.C. (2023). Ocular manifestations of central insulin resistance. Nerual Regen..

[214] Zerbini G., Maestroni S., Vigano I., Mosca A., Paleari R., Gabellini D., Galbiati S., Rama P. (2023). Progressive Thinning of Retinal Nerve Fiber Layer/Ganglion Cell Layer (RNFL/GCL) as Biomarker and Pharmacological Target of Diabetic Retinopathy. Int. J. Mol. Sci..

[215] Elsherbiny N.M., Abdel-Mottaleb Y., Elkazaz A.Y., Atef H., Lashine R.M., Youssef A.M., Ezzat W., El-Ghaiesh S.H., Elshaer R.E., El-Shafey M. (2019). Carbamazepine Alleviates Retinal and Optic Nerve Neural Degeneration in Diabetic Mice via Nerve Growth Factor-Induced PI3K/Akt/mTOR Activation. Front. Neurosci..

[216] Ibán-Arias R., Lisa S., Mastrodimou N., Kokona D., Koulakis E., Iordanidou P., Kouvarakis A., Fothiadaki M., Papadogkonaki S., Sotiriou A. (2017). The Synthetic Microneurotrophin BNN27 Affects Retinal Function in Rats With Streptozotocin-Induced Diabetes. Diabetes.

[217] Iban-Arias R., Lisa S., Poulaki S., Mastrodimou N., Charalampopoulos I., Gravanis A., Thermos K. (2019). Effect of topical administration of the microneurotrophin BNN27 in the diabetic rat retina. Graefe’s Arch. Clin. Exp. Ophthalmol..

[218] Kokkali M., Karali K., Thanou E., Papadopoulou M.A., Zota I., Tsimpolis A., Efstathopoulos P., Calogeropoulou T., Li K.W., Sidiropoulou K. (2025). Multimodal beneficial effects of BNN27, a nerve growth factor synthetic mimetic, in the 5xFAD mouse model of Alzheimer’s disease. Mol. Psychiatry.

[219] LaNoue K.F., Berkich D.A., Conway M., Barber A.J., Hu L.Y., Taylor C., Hutson S. (2001). Role of specific aminotransferases in de novo glutamate synthesis and redox shuttling in the retina. J. Neurosci. Res..

[220] Ola M.S., Alhomida A.S., LaNoue K.F. (2019). Gabapentin Attenuates Oxidative Stress and Apoptosis in the Diabetic Rat Retina. Neurotox. Res..

[221] Kusari J., Zhou S., Padillo E., Clarke K.G., Gil D.W. (2007). Effect of memantine on neuroretinal function and retinal vascular changes of streptozotocin-induced diabetic rats. Investig. Ophthalmol. Vis. Sci..

[222] Wang L., Deng Q.Q., Wu X.H., Yu J., Yang X.L., Zhong Y.M. (2013). Upregulation of glutamate-aspartate transporter by glial cell line-derived neurotrophic factor ameliorates cell apoptosis in neural retina in streptozotocin-induced diabetic rats. CNS Neurosci. Ther..

[223] Gu L., Xu H., Wang F., Xu G., Sinha D., Wang J., Xu J.Y., Tian H., Gao F., Li W. (2014). Erythropoietin exerts a neuroprotective function against glutamate neurotoxicity in experimental diabetic retina. Investig. Ophthalmol. Vis. Sci..

[224] Dionysopoulou S., Wikstrom P., Walum E., Georgakis S., Thermos K. (2024). Investigation of the Effects of a Novel NOX2 Inhibitor, GLX7013170, against Glutamate Excitotoxicity and Diabetes Insults in the Retina. Pharmaceuticals.

[225] Shu X., Zhang Y., Li M., Huang X., Yang Y., Zeng J., Zhao Y., Wang X., Zhang W., Ying Y. (2019). Topical ocular administration of the GLP-1 receptor agonist liraglutide arrests hyperphosphorylated tau-triggered diabetic retinal neurodegeneration via activation of GLP-1R/Akt/GSK3beta signaling. Neuropharmacology.

[226] Boccaccini A., Cavaterra D., Carnevale C., Tanga L., Marini S., Bocedi A., Lacal P.M., Manni G., Graziani G., Sbardella D. (2023). Novel frontiers in neuroprotective therapies in glaucoma: Molecular and clinical aspects. Mol. Asp. Med..

[227] Rossetti L., Goni F., Montesano G., Stalmans I., Topouzis F., Romano D., Galantin E., Delgado-Gonzales N., Giammaria S., Coco G. (2023). The effect of citicoline oral solution on quality of life in patients with glaucoma: The results of an international, multicenter, randomized, placebo-controlled cross-over trial. Graefe’s Arch. Clin. Exp. Ophthalmol..

[228] Prinz J., Prokosch V., Liu H., Walter P., Fuest M., Migliorini F. (2023). Efficacy of citicoline as a supplement in glaucoma patients: A systematic review. PLoS ONE.

[229] Hui F., Tang J., Williams P.A., McGuinness M.B., Hadoux X., Casson R.J., Coote M., Trounce I.A., Martin K.R., van Wijngaarden P. (2020). Improvement in inner retinal function in glaucoma with nicotinamide (vitamin B3) supplementation: A crossover randomized clinical trial. Clin. Exp. Ophthalmol..

[230] De Moraes C.G., John S.W.M., Williams P.A., Blumberg D.M., Cioffi G.A., Liebmann J.M. (2022). Nicotinamide and Pyruvate for Neuroenhancement in Open-Angle Glaucoma: A Phase 2 Randomized Clinical Trial. JAMA Ophthalmol..

[231] Scuteri D., Bagetta G., Nucci C., Aiello F., Cesareo M., Tonin P., Corasaniti M.T. (2020). Evidence on the neuroprotective properties of brimonidine in glaucoma. Prog. Brain Res..

[232] Otsubo M., Sase K., Tsukahara C., Fujita N., Arizono I., Tokuda N., Kitaoka Y. (2024). Axonal protection by combination of ripasudil and brimonidine with upregulation of p-AMPK in TNF-induced optic nerve degeneration. Int. Ophthalmol..

[233] Trento M., Durando O., Lavecchia S., Charrier L., Cavallo F., Costa M.A., Hernandez C., Simo R., Porta, M., for the EUROCONDOR Trial Investigators (2017). Vision related quality of life in patients with type 2 diabetes in the EUROCONDOR trial. Endocrine.

[234] Simo R., Hernandez C., Porta M., Bandello F., Grauslund J., Harding S.P., Aldington S.J., Egan C., Frydkjaer-Olsen U., Garcia-Arumi J. (2019). Effects of Topically Administered Neuroprotective Drugs in Early Stages of Diabetic Retinopathy: Results of the EUROCONDOR Clinical Trial. Diabetes.

[235] Jung K.I., Kim J.H., Han J.-S., Park C.K. (2024). Exploring Neuroprotective Effects of Topical Brimonidine in Experimental Diabetic Retinopathy. In Vivo.

[236] Zuclich J.A., Stolarski D.J. (2001). Retinal damage induced by red diode laser. Health Phys..

[237] Gold G., Giannakopoulos P., Herrmann F.R., Bouras C., Kövari E. (2007). Identification of Alzheimer and vascular lesion thresholds for mixed dementia. Brain.

